# Potential use of text classification tools as signatures of suicidal behavior: A proof-of-concept study using Virginia Woolf’s personal writings

**DOI:** 10.1371/journal.pone.0204820

**Published:** 2018-10-24

**Authors:** Gabriela de Ávila Berni, Francisco Diego Rabelo-da-Ponte, Diego Librenza-Garcia, Manuela V. Boeira, Márcia Kauer-Sant’Anna, Ives Cavalcante Passos, Flávio Kapczinski

**Affiliations:** 1 Bipolar Disorder Program and Laboratory of Molecular Psychiatry, Federal University of Rio Grande do Sul, Porto Alegre, RS, Brazil; 2 Graduation Program in Psychiatry and Department of Psychiatry, Federal University of Rio Grande do Sul (UFRGS), Porto Alegre, RS, Brazil; 3 Department of Psychiatry and Behavioural Neurosciences, McMaster University, Hamilton, ON, Canada; 4 Department of Psychiatry and Behavioral Neurosciences, St. Joseph Health Hamilton, Hamilton, ON, Canada; University of Toronto, CANADA

## Abstract

**Background:**

The present study analyzes the feasibility of text classification to predict individual suicidal behavior. Entries from Virginia Woolf’s diaries and letters were used to assess whether a text classification algorithm could identify written patterns associated with suicide.

**Methods:**

This is a text classification study. We compared 46 text entries from the two months before Virginia Woolf’s suicide with 54 texts randomly selected from Virginia Woolf’s work during other periods of her life. Letters and diaries were included, while books, novels, short stories, and article fragments were excluded. The data was analyzed using a Naïve-Bayes machine-learning algorithm.

**Results:**

The model showed a balanced accuracy of 80.45%, sensitivity of 69%, and specificity of 91%. The Kappa statistic was 0.6, which means a good agreement, and the p-value of the model was 0.003. The area under the ROC curve (AUC) was 0.80. In other words, the model exhibited good performance when used for classifying Virginia Woolf’s diaries and letters.

**Discussion:**

The present study showed the feasibility of a machine-learning model coupled with text to identify individual written patterns associated with suicidal behavior. Our text signature was able to identify the period of two months preceding suicide with a high accuracy. This technique may be applied to subjects with psychiatric disorders by means of data captured from social media, e-mail, among others. The algorithm may then predict a specific outcome and enable early intervention by clinicians.

## Introduction

It has been shown that approximately 90% of subjects who die through suicide are diagnosed with a psychiatric disorder prior to their death [1]. Suicide is particularly worrisome in Bipolar Disorder (BD), given the high prevalence of this disorder and the strong association between suicide and depressive symptoms [2]. In addition, a large cohort study found that among men, the absolute risk of suicide was higher in BD (7.8%) compared to any other psychiatric condition. Among women, BD was associated with the second highest risk, at 4.8%, just below schizophrenia at 4.9% [3]. Furthermore, patients with BD showed twice the rate of suicide risk than patients with Major Depressive Disorder and about 20 to 30 times greater risk than in the general population [4].

It is known that at least 90% of suicide cases occur in people with a psychiatric disorder, with mood disorders being the most prevalent condition in this scenario [1]. In addition, many of the individuals are untreated or inadequately treated [5]. Suicide prediction tools may aid health professionals to identify subjects at-risk for suicidal behavior, thus enabling not only early intervention and personalized care, but also the development of new strategies for suicide prevention. As there are already interventions available that show anti-suicidal effects, such as lithium for mood disorders [6] and clozapine for schizophrenia [7], adequate detection and treatment of these conditions may help reduce suicide rates.

Virginia Woolf was a British novelist who biographers suggest suffered from BD [8]. From 1910 to 1913, Woolf was ill on several occasions [9]. During her life, she made at least three suicide attempts. She received the habitual treatments of the time, such as “rest cure therapy at home,” which consisted of gaining weight, sleeping, and “the resting of the intellect,” which also meant a recommendation not to write [10]. Virginia endured several depressive and manic episodes until she took her life [11] on March 28, 1941 during a depressive episode [10].

Virginia Woolf presented several known risk factors associated with suicide in patients with BD [12] such as early traumatic experiences (sexual abuse), psychotic symptoms, family history of suicide, and a higher number of depressive episodes. Nevertheless, a meta-analysis of 365 studies showed that traditional suicide risk factors are poor predictors of a future suicide attempt [13]. A possible explanation for this finding is that complex patterns of interaction between risk factors are not considered in traditional statistics [14,15]. Moreover, the risk factors identified so far come from group-level results, and do not wield satisfactory predictive results at an individual level [13]. Thus, it is paramount to develop better models to predict suicidal behavior that include these interactions and explore different levels of information. Machine learning, a field of artificial intelligence that focuses on algorithms that can learn from data and then extract patterns to make new assumptions from unseen information, are increasingly being used in behavioral sciences to provide predictive models for clinical practice [16]. Machine learning can handle an enormous amount of data, such as text data, and combine them in nonlinear and highly interactive ways [17]. One important question is whether suicidal behavior is associated with an identifiable writing pattern. Virginia Woolf left a vast written repertoire contained in her diaries, where she wrote freely about her feelings, providing a living record of her past mood states.

Machine-learning studies already published include the use of natural processing language to detect emotions present in suicide notes [18] and classify them [19], to detect suicidality in Twitter activity [20], and to extract a particular emotion in a suicide note sentence [21,22]. Similarly, it appears that electronic health records may also be of value to predict suicidal behavior [13,22]. Besides text classification, previous studies used machine-learning algorithms to predict suicide. For instance, a study reported a clinical signature by using a relevance vector machine to identify suicidality in patients with mood disorders [2]. The most relevant predictor variables in distinguishing attempters from non-attempters were previous hospitalizations for depression, lifetime history of psychosis, cocaine dependence, and post-traumatic stress disorder comorbidity [2]. Another study that used machine-learning algorithms showed that individual unrest, personal satisfaction, and reasons for living are the variables most associated with suicidality [23]. They concluded that these variables could be used to create an actionable assessment tool that may identify individual risk and protective factors [23].

The present study aims to analyze whether text classification coupled with machine-learning algorithms can predict unfavorable outcomes, such as suicide, using the written records of a single individual. In order to test this hypothesis, we used the content of Virginia Woolf’s diaries and letters to assess whether there is a text signature in her writings prior to her suicide.

## Methods

This is a text classification study with a machine-learning approach. We used a Naïve Bayes algorithm. It is worth mentioning that Naïve Bayes is a Bayesian method that estimates the probability of an event’s occurrence [17]. Although Naïve Bayes is not the only machine-learning method that utilizes Bayesian statistics, it is one of the most common. This is particularly true for text classification, where it has become the *de facto* standard [17]. For instance, this algorithm is commonly used to classify e-mails in spam or ham messages [17]. This algorithm was selected because 1) it requires relatively few examples for training, but also works well with very large numbers of examples; 2) it provides the estimated probability for a prediction; and 3) it is very effective and performs well with noisy data. Therefore, by using Naïve Bayes, we compared Virginia Woolf’s texts written over the 60 days before her death versus texts written outside this period to identify a signature associated with suicide. We included letters and diaries written by Virginia Woolf. We excluded books, novels, short stories, and article fragments.

### Data processing and machine-learning approach

In the present study, we used the R program (version 3.3.1) and the R packages called *tm* and *e1071*. We first typed Virginia Woolf’s letters and diaries into an Excel spreadsheet. The cutoff of 60 days before suicide was used to label a specific text as related to suicide or not. The period of 60 days was arbitrarily defined, but was based on 1) Virginia Woolf’s behavior prior to killing herself, and 2) the need to establish a target time frame. It seems that Virginia Woolf decided to attempt suicide at least several days before she finally took her own life. One week before she died [11], she arrived home soaking wet after having survived a suicide attempt. According to Woolf’s husband, she looked ill and shaken but she told him that she had slipped into a dyke [24]. Later on, Virginia Woolf filled her overcoat pockets with heavy stones and headed to the River Ouse. Regarding methodological issues, there is no sample size calculation for a machine-learning model because the algorithm benefits from larger samples. Therefore, 1) we arbitrarily used a 60-day period to provide an adequate number of observations; 2) we imported the dataset into the R program and converted it into a representation called “bag-of-words,” which ignores word order and simply provides a variable indicating whether the word appears at all; 3) we standardized the texts to use only lowercase characters and removed additional white spaces, numbers, and punctuation by using the *tm* package; 4) we split the texts into individual components through a process called tokenization. In this process, we created a data structure called document term matrix (DTM), a sparse matrix in which rows indicate documents (i.e. Virginia Woolf’s letters and diaries) and columns indicate terms (the frequency of each word that appeared in each text); 5) we randomized our data and then divided it into training and test datasets; 6) we filtered our training and test DTM to include only the words appearing in at least five documents; 7) we applied the Naïve Bayes algorithm in the training dataset to build the model by using the *e1071* package. The algorithm used the presence or absence of words to estimate the probability that a given document would be related to suicide; 8) we evaluated the model performance by testing its predictions on unseen documents in the test dataset. Accordingly, we used the model to produce predictions and then to compare them to the true values. The model’s performance in the test dataset was assessed with the AUC, accuracy, sensitivity, specificity, positive predictive value, and negative predictive value. The Cohen’s kappa index is used to control for the accuracy of a random classifier as measured by the expected accuracy. Other analysis was performed using other parameters (S1–S9 Figs).

#### Word cloud

For this step, we used the R package called *word cloud*. A word cloud is a way to visually depict the frequency with which words appear in text data. The cloud is composed of words scattered somewhat randomly around the figure. Words appearing more often in the text are shown in a larger font, while less common terms are shown in smaller fonts. Of note, this type of figure has grown in popularity recently, since it provides a way to observe trending topics on social media websites. In the present study, we generated two word clouds, one with the texts written 60 days before Virginia Woolf’s suicide and another with the texts written outside this period to analyze the trending topics on her work during these periods. We selected 20 as the minimal frequency parameter. The minimal frequency parameter specifies the number of times a word must appear in the corpus before it will be displayed in the cloud.

## Results

We included 46 texts written 60 days before suicide and 54 texts written outside this period (S1 File). Of note, we selected almost the same number of letters and diaries in each group in order to avoid the so-called class imbalance problem, which occurs when one outcome is more frequent than the other [2].

### Machine-learning model performance

**Fig 1:**
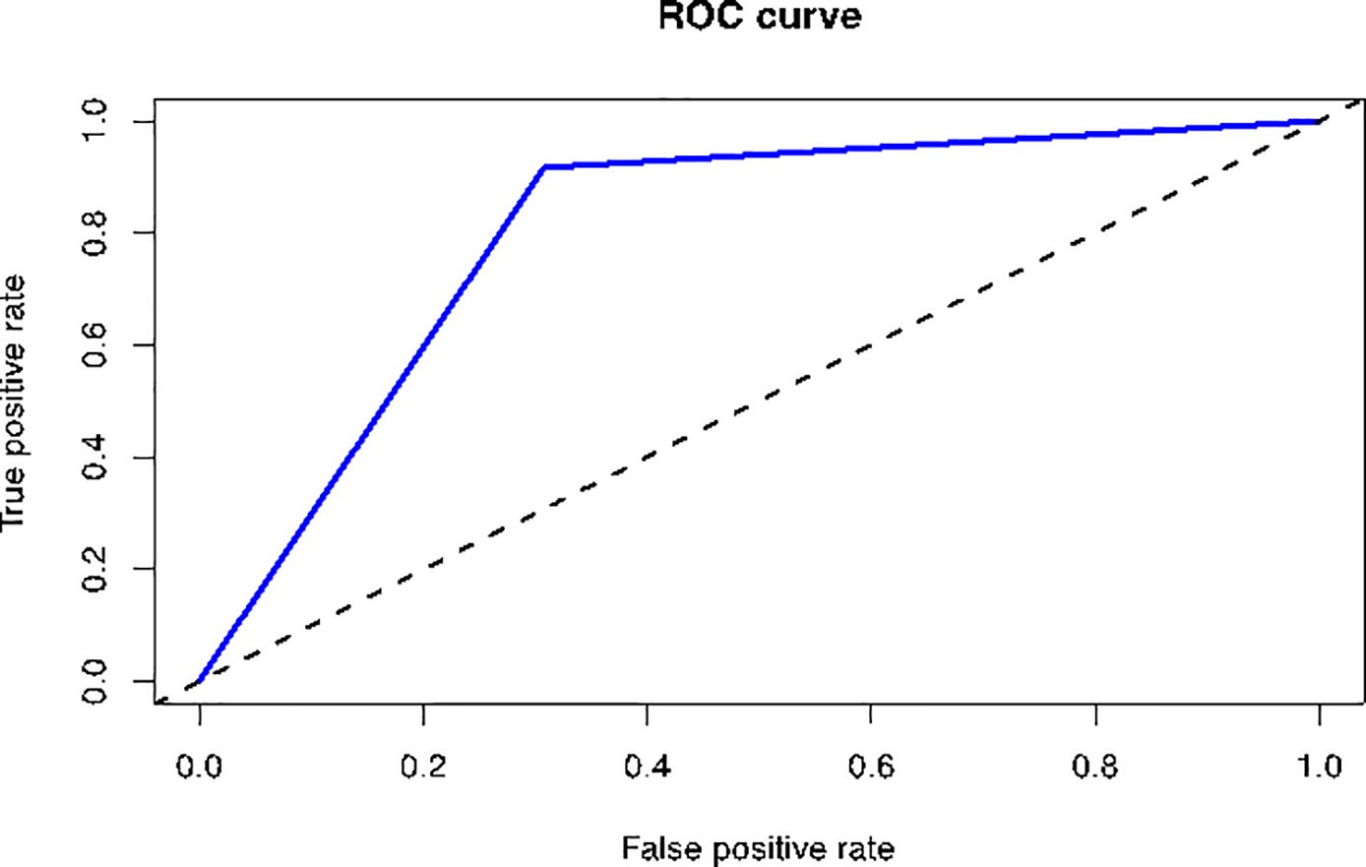
Receiver operating characteristic curve of the text classification model.

The model showed an accuracy of 80.00% with a kappa index of 0.6 to detect Virginia Woolf’s suicide from the included writings. Sensitivity and specificity were 69.23 and 91.67%, respectively, with a balanced accuracy of 80.45%, controlling for the accuracy of a random classifier as measured by the expected accuracy. The p-value was 0.003 for the text classification model. Furthermore, the positive predictive value was 90.00% and the negative predictive value was 73.33% (Fig 1).

#### Word cloud

**Fig 2:**
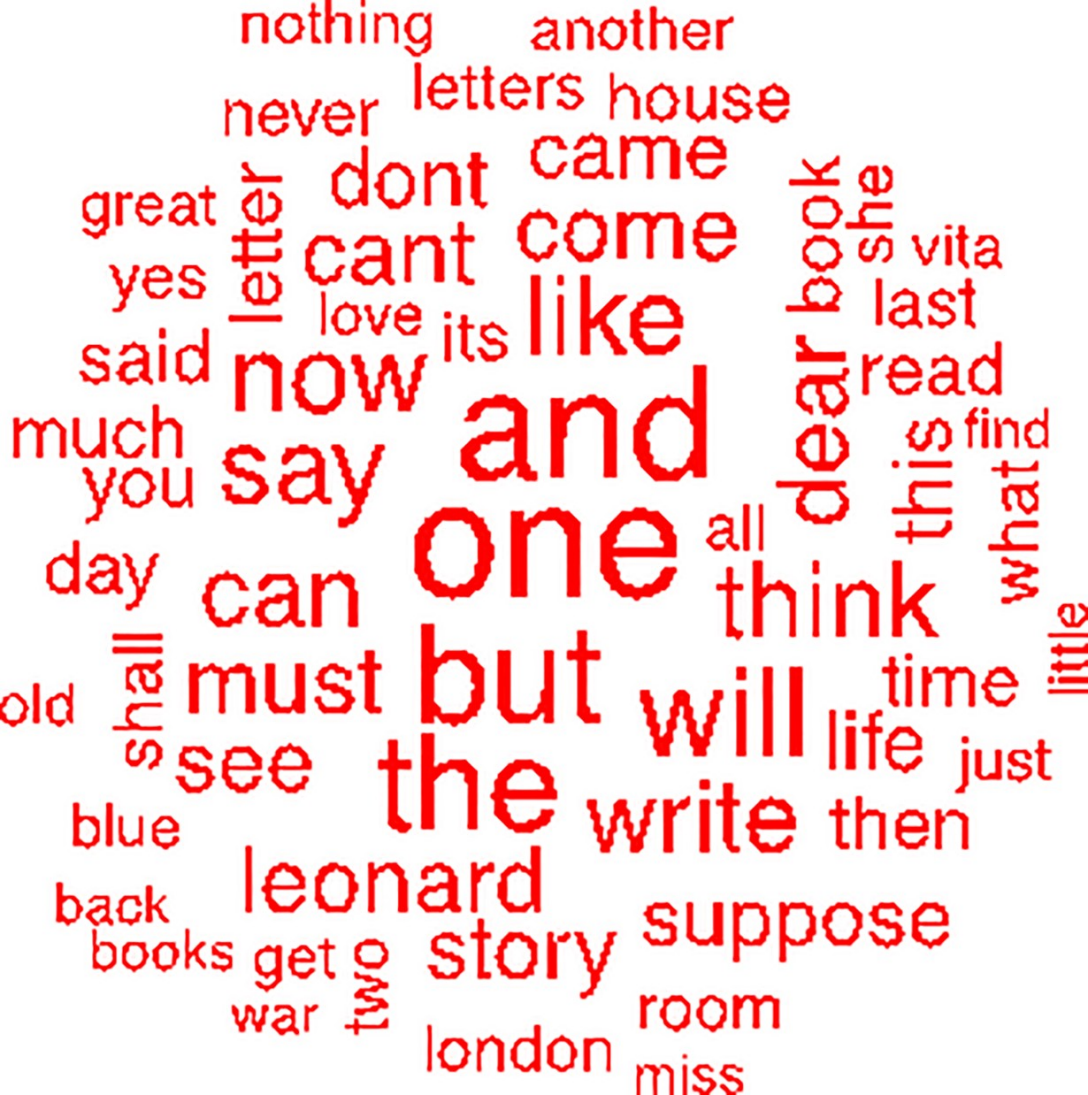
Word cloud of words written in the last 60 days before Virginia Woolf’s suicide.

Fig 2 shows the word cloud for the letters and diaries associated with Virginia Woolf’s suicide (S1 Table). The words that appear exclusively in the suicide word cloud are “blue,” “books,” “house,” “miss,” “she,” “suppose,”“yes,” “you,” “Vita,” and “war” (S2 Table).

**Fig 3:**
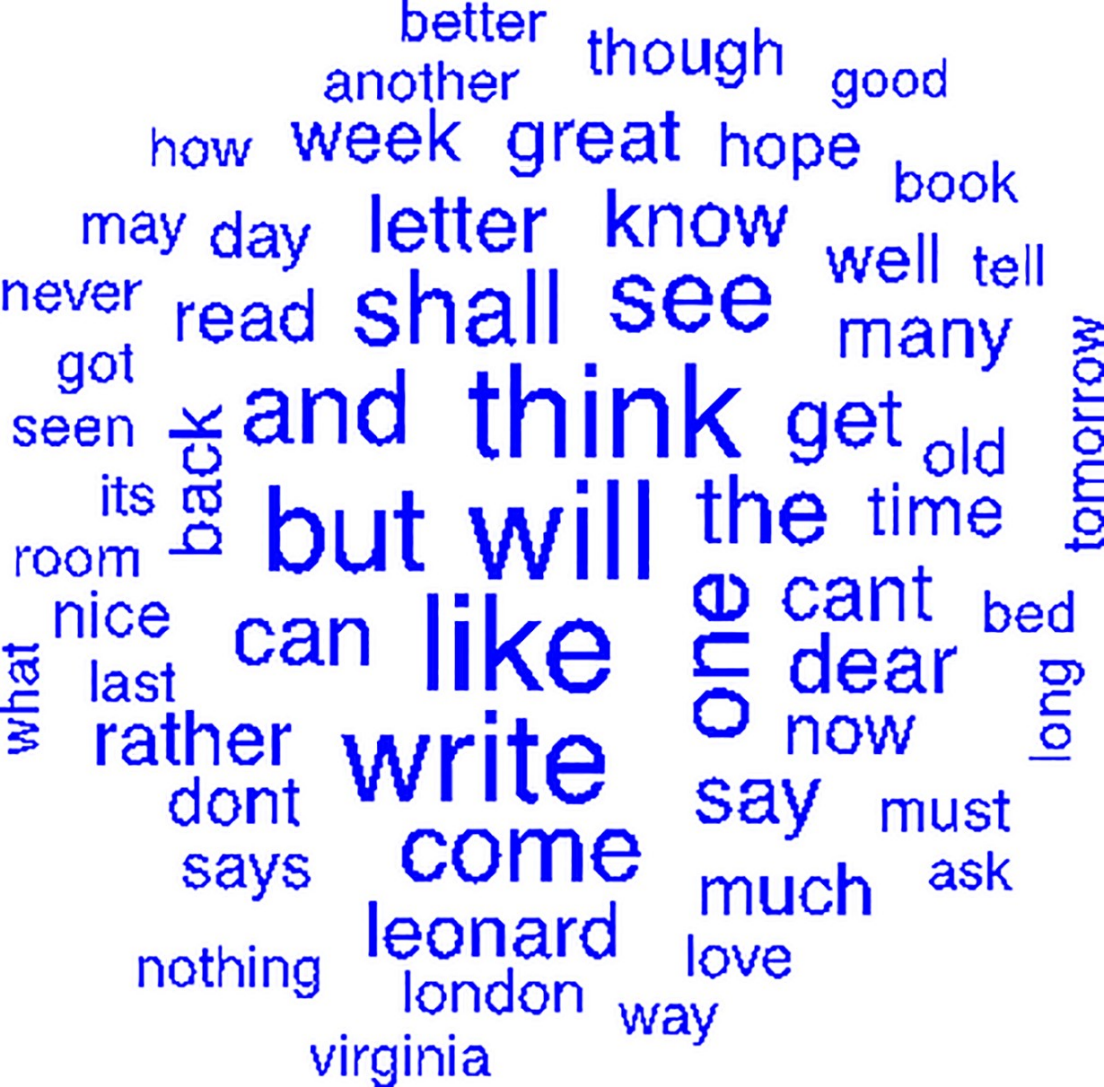
Word cloud of words written in randomly selected periods, outside the two months prior to Virginia Woolf’s suicide.

Fig 3 shows the word cloud for those words outside the suicide period (S3 Table). The words that appear exclusively in the word cloud written out of the period leading up to Virginia Woolf’s suicide are “ask,” “better,” “good,” “got,” “hope,” “how,” “know,” “long,” “many,” “may,” “nice,” “rather,” “says,” “tomorrow,” “Virginia, and “well” (S2 Table).

Furthermore, there are common words in the two clouds such as “book,” “write,” “letter,” “can,” “come,” “Leonard,” “like,” “shall,” “must,” and “will” but they are more frequent in the non-suicide word cloud than in the suicide word cloud. However, “never” and “nothing” appear more frequently in the non-suicide word cloud (S4 Table).

## Discussion

The present study is the first to analyze the feasibility of a machine-learning model coupled with text data to identify a pattern in writings previous to suicide, herein applied by using the diaries and letters of a novelist. Our text classification signature was able to predict suicide with an accuracy of 80%, sensitivity of 69.23%, and specificity of 91.67%. This shows the potential impact of this kind of approach for identifying linguistic markers in the letters/diaries indicating the deterioration in the writer’s mental health, which would lead to suicide. It is worth mentioning that a previous study attempted to predict suicide based on text data, but not using data from the subject who died by suicide [11,25]. The authors developed a linguistic-driven prediction model with the clinical notes taken from medical records and achieved an accuracy of 65% to predict suicide risk [25]. Another report generated datasets of single keywords and multi-word phrases and constructed prediction models using a machine-learning algorithm based on a genetic programming framework [26]. Text mining is able to solve complex tasks such as identifying spam messages in e-mails, protein interaction networks, human emotions, and customer attrition [17,27–30]. Moreover, other studies used clinical notes to predict suicidal behavior, utilizing machine-learning algorithms [22,25].

We performed analysis with term frequency of more than three or four times. All results showed a balanced accuracy of 80%. However, when the model was performed excluding the words “and,” “one,” “but,” and “the,” the balanced accuracy reduced to 59% (sensitivity: 94%; specificity: 25%). In other words, the performance of the algorithm improves with inclusion of the terms “and,” “one,” “but,” and “the” (S1–S9 Fig).

The words that appear only in the non-suicide word cloud are “ask,” “better,” “good,” “got,” “hope,” “how,” “know,” “long,” “many,” “may,” “nice,” “rather,” “says,” “tomorrow,” “Virginia,” and “well.” The word “better” may reflect her mood at that time; “good,” “got,” and “nice” all have a positive meaning. “Well,” “hope,” and “tomorrow” also have a positive meaning and could show a sense of hope. When we look at the common words, that is, the words that appear in both word clouds, we see that some of them presented a higher frequency in the non-suicide word cloud, such as: 1) “book,” “write,” and “letter”–the higher frequency may occur because that was a period when Virginia used to write more; 2) “like” and “love”–words that have a positive meaning; and 3) “shall,” “must,” “will,” and “can”–which may express possibilities, things to do, or even better, self-confidence. Conversely, words with a negative meaning, such as “never” and “nothing” also appeared with higher frequency in the non-suicide word cloud. The model uses frequency tables of several simultaneous words (whether they appear or not in a specific letter in the present study) to learn the data. In view of this, those interpretations are subjective and were made by the authors, not by the model.

Cognitive distortions frequently occur during mood episodes [29,30] and are associated with risk of suicide [31]. Some words with negative meanings, such as “war,” “blue,” and “miss” appear only in the suicide word cloud and are possibly related to Virginia Woolf’s depression (Fig 2). In this context, negative words could represent her thoughts of lack of efficacy, self-criticism, worthlessness, nostalgia, melancholy, and mainly hopelessness. As the model analyzes frequency of words and not whole sentences, it is not possible to assess whether negative words were used in a negative context.

In the present study, we showed that machine learning may be used to analyze text data from a single individual to create a semantic signature associated with the period where suicide took place. This proof-of-concept study illustrates the potential of machine-learning techniques coupled with text analysis to identify avoidable outcomes such as suicide. We therefore hypothesize that real-time machine learning, a method that creates a predictive model as the data is being created, may be applied to patients with psychiatric disorders. Such a method holds the potential to predict unfavorable outcomes, by collecting data from smartphones, internet use, and e-mail messages, among others. For example, when an adverse event is flagged as an outcome of interest by the clinician (e.g. suicide, mood episode relapse, or psychotic symptoms) the algorithm can analyze the days preceding output to find patterns that will enable predictions of when these events are likely to reoccur. This may improve the patient’s assessment, enabling early intervention and providing real-time insights for clinicians on mood status and suicide risk. Moreover, such models could be personalized to a single patient level, creating an artificial intelligence loop that adapts as data is collected over time.

Our study has some limitations. First, this is a single individual study where our sample included a total of 100 texts in the machine-learning model. Second, we arbitrarily defined the period of 60 days prior to the suicide for the analysis. As this is a unique proof-of-concept study, it is impossible to estimate what would be the ideal observation time. It is even possible that the pattern of change and speed of change may be relevant in the analysis of text production as a correlate of behavior. Third, it is not clear whether the signature that we found is representative of suicide or whether it is associated with one unique depressive episode or thoughts related to this unique period in the life of Virginia Woolf. However, it is probably impossible to disentangle one thing from another. Fourth, it is not possible to rule out that her written pattern change in the days before suicide was due to other factor(s) and not necessarily her suicidal behavior. However, in catastrophic outcomes such as suicide, high sensitivity may be desirable even if specificity is lost. Also, it is not possible to confirm that letters outside the observation period of 60 days before suicide were representative of a period free of symptoms. Furthermore, it is worth mentioning that this text signature is specific for one single individual and may not be useful outside the single-individual context.

The present study analyzed the content of Virginia Woolf's diaries and letters and identified a pattern unique to the period of two months prior to her suicide. Future studies in the field of suicide prevention and predictive analysis will determine the usefulness of text classification in the prediction of suicidal behavior in individuals at risk.

## Supporting information

S1 Fig Receiver Operating Characteristic curve of the text classification model with the criteria of at least 3 appearances. Results with the criteria of at least 3 appearances. Balanced Accuracy: 0.80. Sensitivity: 0.69. Specificity: 0.91. P-Value: 0.003. Kappa: 0.6. AUC: 0.80.
(TIF)

S2 Fig Word cloud of words written in the last 60 days before Virginia Woolf’s suicide with the criteria of at least 3 appearances. (TIF)

S3 Fig Word cloud of words written in randomly selected periods, outside the two months prior to Virginia Woolf’s suicide with the criteria of at least 3 appearances. (TIF)

S4 Fig Receiver Operating Characteristic curve of the text classification model with the criteria of at least 4 appearances. Results with the criteria of at least 4 appearances. Balanced Accuracy: 0.80. Sensitivity: 0.76. Specificity: 0.83. P-Value: 0.003. Kappa: 0.6. AUC: 0.80.
(TIF)

S5 Fig Word cloud of words written in the last 60 days before Virginia Woolf’s suicide with the criteria of at least 4 appearances. (TIF)

S6 Fig Word cloud of words written in randomly selected periods, outside the two months prior to Virginia Woolf’s suicide with the criteria of at least 3 appearances. (TIF)

S7 Fig Receiver Operating Characteristic curve of the text classification model without words "and,” “one,” “the,” “but”. **Results without words "and,” “one,” “the,” “but”.** Balanced Accuracy: 0.59. Sensitivity: 0.94. Specificity: 0.25. P-Value: 0.42. Kappa: 0.22. AUC: 0.59.
(TIF)

S8 Fig Word cloud of words written in the last 60 days before Virginia Woolf’s suicide without words "and,” “one,” “the,” “but”. (TIF)

S9 Fig Word cloud of words written in randomly selected periods, outside the two months prior to Virginia Woolf’s suicide without words "and,” “one,” “the,” “but”. (TIF)

S1 File Dataset with Virginia Woolf’s texts analyzed. (PDF)

S1 Table Words written in the last 60 days before Virginia Woolf’s suicide. (PDF)

S2 Table Different words written in the last 60 days before Virginia Woolf’s suicide compared to outside the 60 days prior to Virginia Woolf’s suicide. (PDF)

S3 Table Words written outside the 60 days prior to Virginia Woolf’s suicide. (PDF)

S4 Table Common words written in the last 60 days before Virginia Woolf’s suicide. (PDF)
