# Correction: Potential use of text classification tools as signatures of suicidal behavior: A proof-of-concept study using Virginia Woolf's personal writings

**DOI:** 10.1371/journal.pone.0207963

**Published:** 2018-11-16

**Authors:** Gabriela de Ávila Berni, Francisco Diego Rabelo-da-Ponte, Diego Librenza-Garcia, Manuela V. Boeira, Márcia Kauer-Sant’Anna, Ives Cavalcante Passos, Flávio Kapczinski

The sixth author’s name is incorrectly abbreviated in the citation. The correct citation is: de Ávila Berni G, Rabelo-da-Ponte FD, Librenza-Garcia D, V. Boeira M, Kauer-Sant’Anna M, Passos IC, et al. (2018) Potential use of text classification tools as signatures of suicidal behavior: A proof-of-concept study using Virginia Woolf’s personal writings. PLoS ONE 13(10): e0204820. https://doi.org/10.1371/journal.pone.0204820
