# Supplementary figures and images for: Potential use of text classification tools as signatures of suicidal behavior: A proof-of-concept study using Virginia Woolf’s personal writings
Source: PLoS One. 2018 Oct 24;13(10):e0204820. doi: 10.1371/journal.pone.0204820 (PMC6200194; doi:10.1371/journal.pone.0204820)

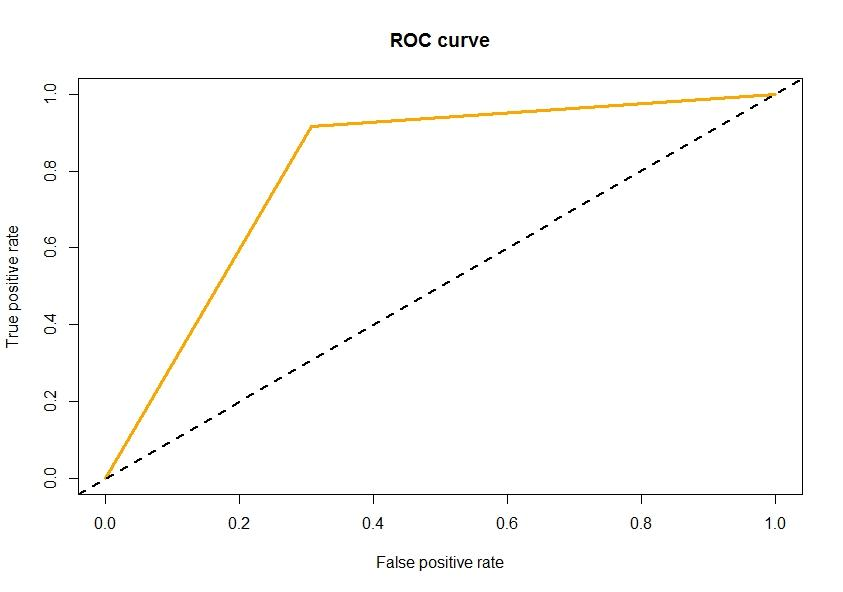

Supplement: S1 Fig — Results with the criteria of at least 3 appearances. Balanced Accuracy: 0.80. Sensitivity: 0.69. Specificity: 0.91. P-Value: 0.003. Kappa: 0.6. AUC: 0.80. (TIF) [file pone.0204820.s001.tif]

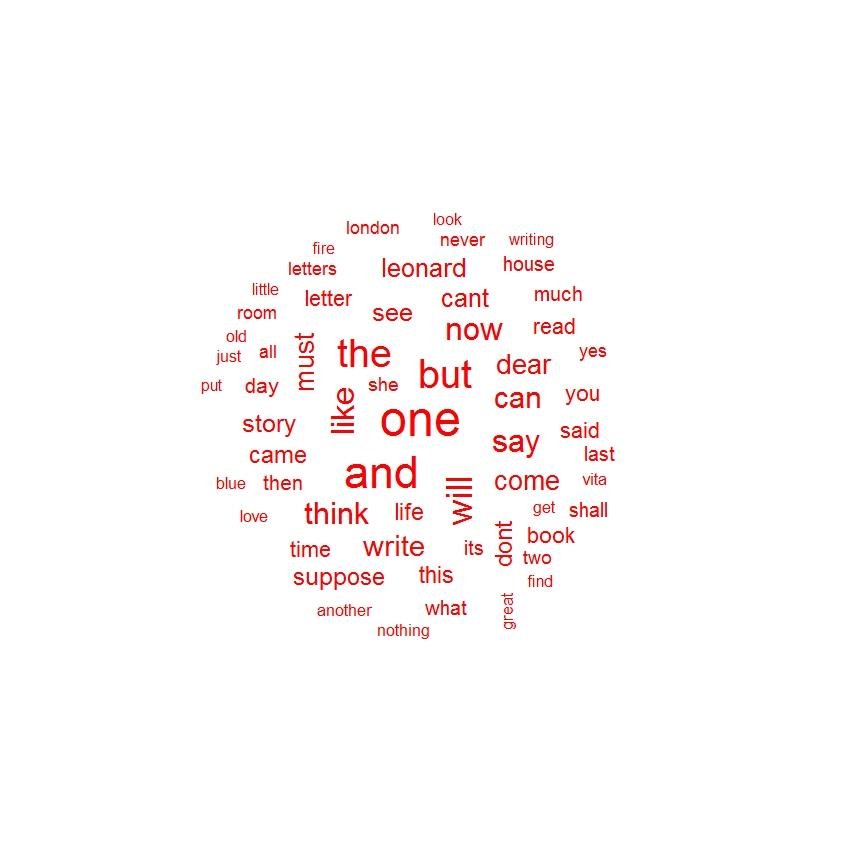

Supplement: S2 Fig — (TIF) [file pone.0204820.s002.tif]

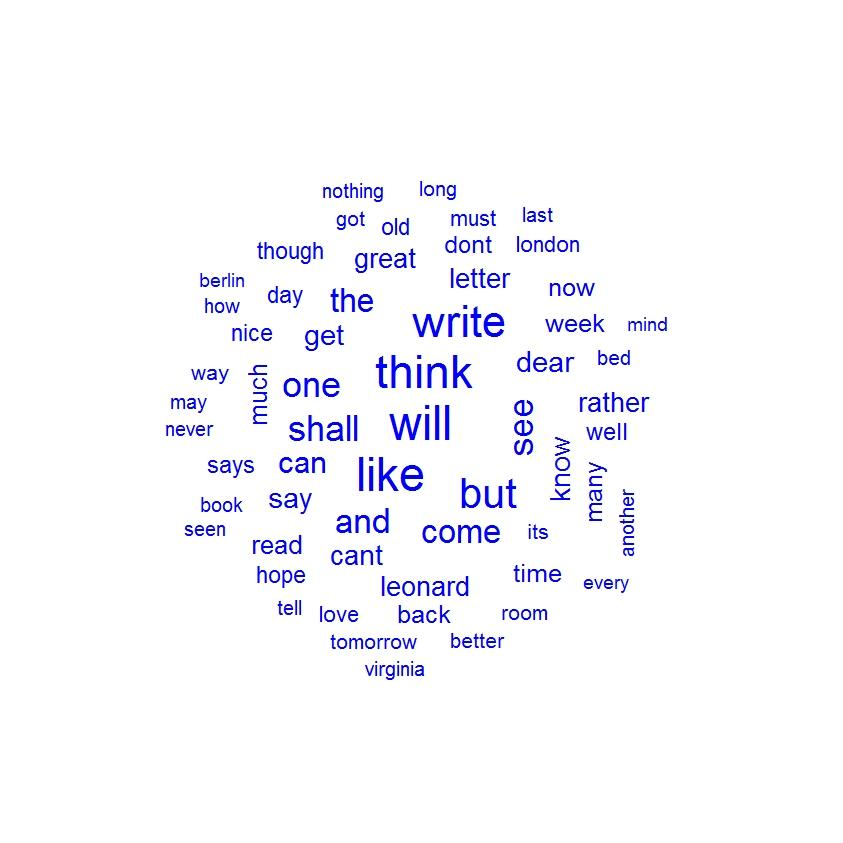

Supplement: S3 Fig — (TIF) [file pone.0204820.s003.tif]

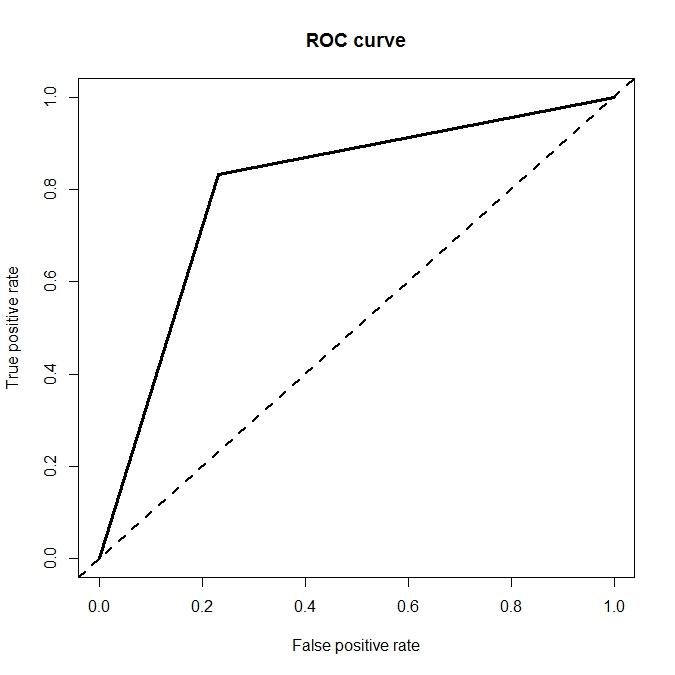

Supplement: S4 Fig — Results with the criteria of at least 4 appearances. Balanced Accuracy: 0.80. Sensitivity: 0.76. Specificity: 0.83. P-Value: 0.003. Kappa: 0.6. AUC: 0.80. (TIF) [file pone.0204820.s004.tif]

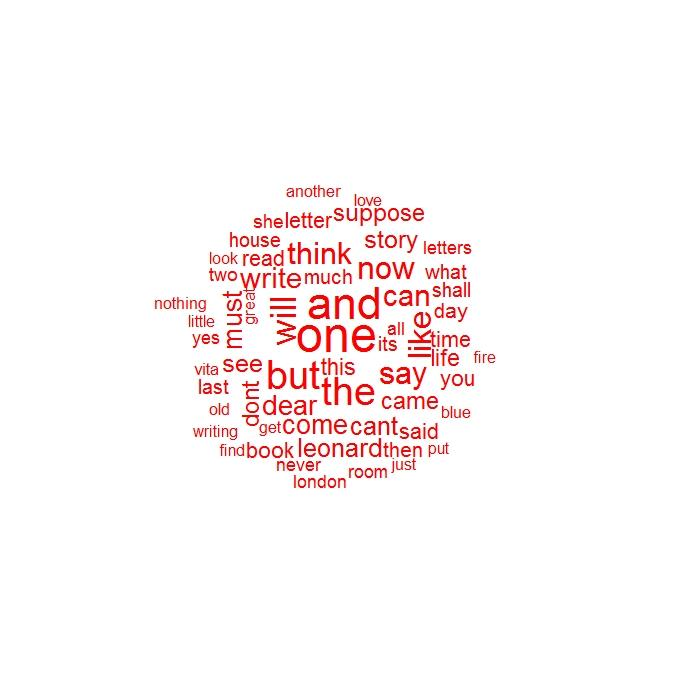

Supplement: S5 Fig — (TIF) [file pone.0204820.s005.tif]

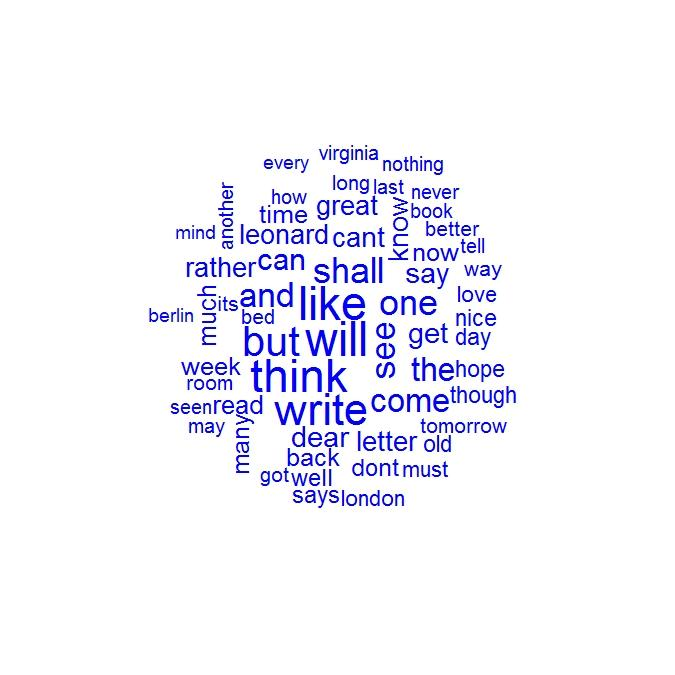

Supplement: S6 Fig — (TIF) [file pone.0204820.s006.tif]

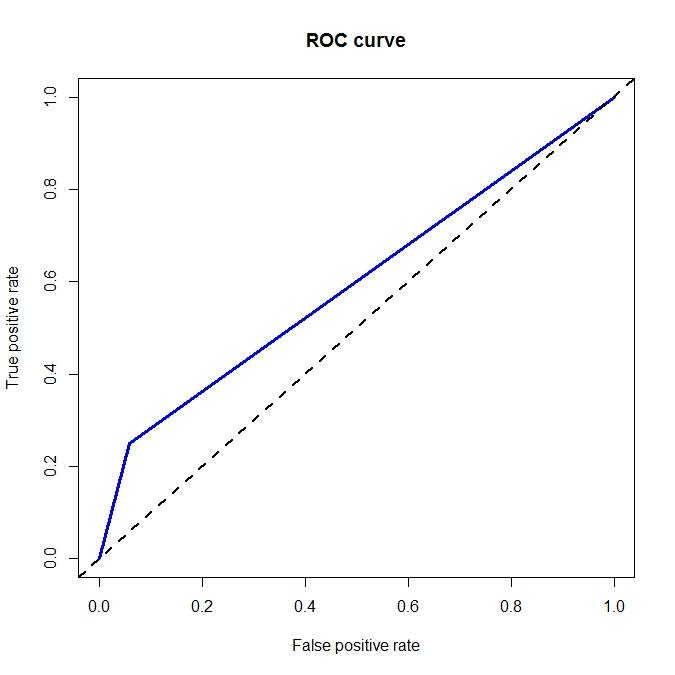

Supplement: S7 Fig — Results without words "and,” “one,” “the,” “but”. Balanced Accuracy: 0.59. Sensitivity: 0.94. Specificity: 0.25. P-Value: 0.42. Kappa: 0.22. AUC: 0.59. (TIF) [file pone.0204820.s007.tif]

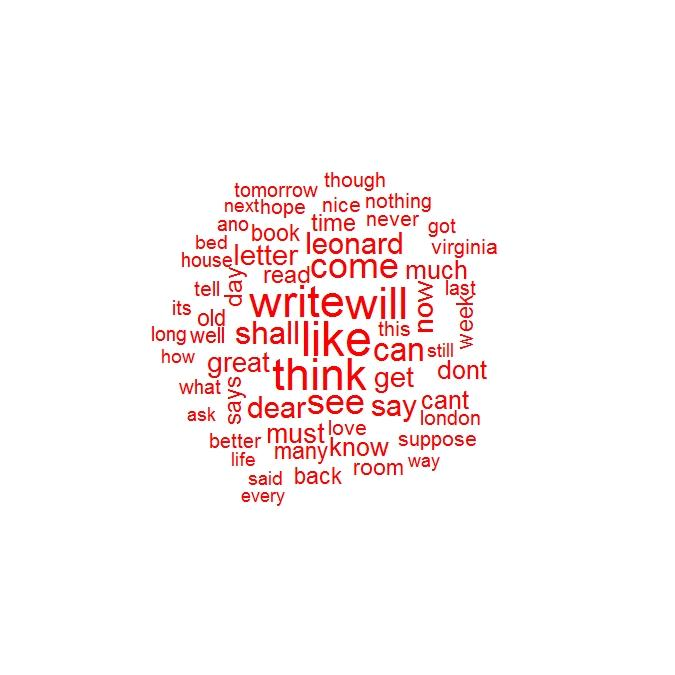

Supplement: S8 Fig — (TIF) [file pone.0204820.s008.tif]

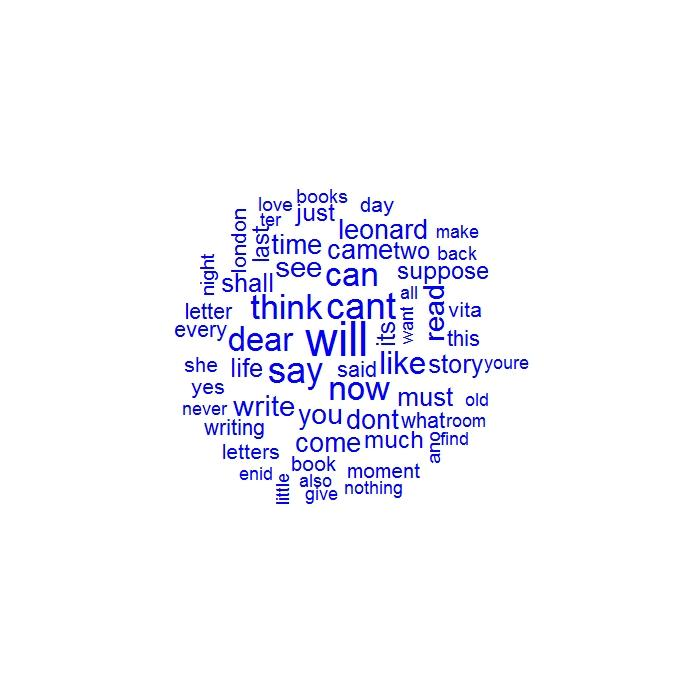

Supplement: S9 Fig — (TIF) [file pone.0204820.s009.tif]
