# Supplementary material for: Potential use of text classification tools as signatures of suicidal behavior: A proof-of-concept study using Virginia Woolf’s personal writings
Source: PLoS One. 2018 Oct 24;13(10):e0204820. doi: 10.1371/journal.pone.0204820 (PMC6200194; doi:10.1371/journal.pone.0204820)
Supplement: S1 File — (PDF) [file pone.0204820.s010.pdf]

Dataset with Virginia Woolf's texts analyzed.

ID#	Diary=0 ; Letter=1	Date	Date of Suicide	Days	Text	Outcome (60 days before suicide=1; outside this period=0)
1	1	04/01/1941	28/03/1941	83	Dearest Sibyl,<br>Of course you have only to suggest any<br>Friday. Not, if you will take my advice, in<br>January. Our hearts are warm, but oh<br>the cold here! Driving snow; downs<br>white; birds frozen; and my hand a<br>mere claw. But in February? As I say,<br>suggest it; but you know it is a bare<br>barn, this house, your blood is on your<br>own head if you come. But I will fry you<br>an egg, and we can crouch over the fire.No Desmond; no Moore. The old<br>Wolves huddle like rooks alone on their<br>tree-top.<br>My only boast is that Margot<br>has given me a statue.<br>Yrs Virginia	0
2	1	09/01/1941	28/03/1941	78	Dear Octavia,<br>Oh dear now you have telephoned,<br>and I was just about to write. We cant<br>come to the concert, as Leonard<br>lectures, and theres the Blackout and<br>the chairs to see to.<br>I am sorry. Think of eating Turkey!<br>and I want to continue the argument<br>the very one-sided argument; books v.<br>cream. I dont see how you can brave it<br>out. Nothing we both ever to the end write can outweigh your milk and cream<br>at this bitter and barren moment.<br>Besides, having some to spare, I gave<br>Louie a jug; and so the Everest family<br>bless your name, having porridge for<br>breakfast. I will keep the boxes. The<br>cartons came unscathed.<br>Yesterday I had a long long letter<br>from Rachel Dyce Sharp, which I will<br>show you when you come. I think the<br>woman is whirling raving mad<br>however. This hand does not shake from book<br>hugging, but from rage. Louie being<br>gone to a funeral, I cooked lunch: and<br>the rice floored me. Thats why I rage,<br>and am now consulting a cookery book.So how am I to write your book?<br>My father has been done already. F. W.<br>Maitland.<br>But I am too rice-infested to make any<br>sense. So forgive; and I am so sorry I	0

3	1	10/01/1941	28/03/1941	77	My dear Elaine, aged about 9,<br>The Blue Cat, whose name is Peat,<br>has asked me to write you a letter as he<br>has not yet learnt how. Well he has a<br>fine story to tell you. When I that is<br>Virginia came into the hall yesterday I<br>saw Peat playing with my glove.<br>Suddenly the glove began to whistle.<br>Then I looked and saw the fingers<br>move. Then I looked again and saw it<br>was a Bat. Leonard! Leonard! I shrieked. The Cat has caught a bat!!<br>He thought it was a joke, so he did not<br>come. He was sawing up wood. Then I<br>got a flower pot and put it over the Bat.<br>But, when Leonard came, the bat was<br>dead. So Peat ate him for his supper.<br>Then we had a great flood, because a<br>bomb burst the river Bank. And we saw<br>all the moles swimming for dear life,<br>only their paws are so short compared<br>with their bodies they did not get far.<br>Then three great hares came and sat in<br>the road. Now the flood is gone. All the<br>marsh is frozen, and I can see three<br>black spots which are really three red<br>cows or hares.<br>Have you had any adventures? I wish you would write and tell me and I will<br>tell Peat. He likes verse better than<br>prose. He and Sally are curled up on<br>the mat to keep each other warm. Only	0
4	1	12/01/1941	28/03/1941	75	Well, well, how time passes. Did I ever<br>thank you for your offer of a £10 note,<br>which was to turn into a car, and the<br>car was to take me to Woking, etc etc<br>No, my sudden financial anguish was<br>only due to doing the years accounts<br>with Leonard, Like my father, I can<br>always conjure up bankruptcy. But<br>unlike my father Leonard has no<br>money complex. So we can rub along<br>cutting a servant, cutting clothes; but otherwise not encroaching on capital<br>yet. Oh yes, I can write: I mean I have a<br>fizz of ideas. What I dread is bottling<br>them to order. Didn't we start the<br>Hogarth Press 25 years ago so as to be<br>quit of editors and publishers? Its my<br>nightmare, being in their clutches: but a<br>nightmare, not a sane survey.<br>I am interested that you cant write<br>about masturbation. That I understand.<br>What puzzles me is how this reticence<br>co-habits with your ability to talk<br>openly magnificently, freely about, say<br>H.B. I couldn't do one or the other. But<br>as so much of life is sexual, or so they<br>say, it rather limits autobiography if<br>this is blacked out. It must be, I suspect, for many generations, for women; for<br>its like breaking the hymen, if thats the<br>membrane name, a painful	0

5	1	18/01/1941	28/03/1941	69	Dear Philip,<br>I am so glad that you enjoyed my life<br>of Roger Fry. I wish you had known<br>him, he was far the most exciting<br>person to meet I have ever known. He was<br>always bubbling with new ideas and<br>adventures, even when the last one had<br>gone smash, like the Omega. I was glad<br>to be able to rescue one of his blue<br>Omega plates from the ruins of<br>Mecklenburgh Square. We have had to move all our furniture down here, as<br>after a landmine broke all the windows<br>for a second time, our flat became<br>uninhabitable. So we are now without a<br>house in London and live entirely here;<br>with our books and beds boarded out<br>in various rooms in the village.<br>I am glad you are in Edinburgh to<br>study medicine, and not London,<br>which is a melancholy place now. Last<br>week I walked through the ruins of the<br>Temple. If you come to Sussex I hope<br>you will visit us.<br>Please remember us both to your<br>mother.<br>yrs Virginia Woolf	0
6	1	19/01/1941	28/03/1941	68	I must buy some shaded inks,<br>lavenders, pinks violets, to shade my<br>meaning. I see I gave you many wrong<br>meanings, using only black ink. It was a<br>joke, our drifting apart. It was serious,<br>wishing you would write. It was not true that<br>I disliked Hilda Matheson. I only felt<br>What? Something opaque,<br>pulverising: my fault, as much as hers.<br>And one pang of wild jealousy seized<br>me, inopportunely, dining at Sibyls. No, no, I must buy my coloured inks. As<br>for Irene; yes: a certain discomfort; as if the<br>past rose between us. I was sorry for<br>one of her trophies a nice young man<br>she met at my house, at once captivated<br>then, I thought, dropped; whereupon<br>he was killed in the war. But no: I must<br>get my coloured inks. She never<br>bedded, only waved flags from her<br>castle tower: and it was a little<br>exacerbating when my brother went off<br>at a gallop. Thats all.<br>The slide arrived safe. But Miss<br>Gardner (who is had a story accepted by<br>Horizon and is thus in the 7th Heaven)<br>says we must get a Lantern. The Epidia-<br>scope (Greek for looking through and over, I think) wont take slides only rung<br>from the top? A sudden thaw: a<br>spring day; and crocuses out in a pot.<br>Potto and V.	0

7	1	23/01/1941	28/03/1941	64	Dear Sir,<br>Your letter of 21 January astonishes<br>me. Three months ago you wrote to me<br>saying that the American office had<br>cabled to you clamouring for a story<br>from me. I was not prepared to submit<br>a story unless commissioned and you<br>then wrote to me on October 25th<br>saying that it would definitely be a commission from America and if you<br>can let me have a story for them as soon<br>as possible we would use it ourselves<br>this side in the next issue to press. You<br>acknowledged receipt of the story on<br>November 4, nearly three months ago.<br>Since that date I have heard nothing<br>from you, my letters remained<br>unanswered, and the story was not<br>used in your next issue. You now write<br>me a letter from which I gather that<br>you propose, without apology, to<br>repudiate your agreementY<br>ours faithfully	0
8	1	25/01/1941	28/03/1941	62	I never thanked you, dear me, for the<br>receipts, recipes which I have laid in<br>my drawer. As for Riding Hoods<br>basket, that is our bi-weekly miracle:<br>really twice a week we have a festival at<br>your expense, and Louie has porridge<br>for breakfast.<br>This damp dismal day its like a sun<br>in the fog. If I cant write, I can eat. As<br>for writing, its a washout.<br>Should you be our way, look in: but this is only by way of thanks, in a rush<br>and a hurry.Yr V. W.<br>Note the envelope, bombed stock from<br>a lawyers office bought cheap in<br>Chancery Lane.<br>Sussex	0

9	1	25/01/1941	28/03/1941	62	My dear Shena,<br>I was so glad to get your letter and<br>write this by way of giving you an<br>excuse. if you want one, to write<br>again. I had enough imagination to suppose<br>that you'd be in the thick of it. And I<br>rather envy you. It seems a little futile<br>to boil with rage as I do about twice a<br>week, in these marshes. This morning<br>it was the soldiers saying women were turning them out of their jobs. The<br>human race seems to repeat itself<br>insufferably. I should like to know<br>about rates I have a personal interest<br>now, fighting the Foundlings about our<br>wrecked flat. And Leonard says I am to<br>tell you how much he admired your<br>pamphlet on Education. This is<br>incoherent, but I am trying to light a fire,<br>this damp day, of green wood.<br>No, I dont see whats to be done<br>about war. Its manliness; and manliness<br>breeds womanliness, both so hateful. I<br>tried to put this to our local labour<br>party: but was scowled at as a<br>prostitute. They said if women had as<br>much money as men, they would enjoy themselves: and then what about the<br>children? So they have more children;<br>more wars; and so on. This is not a<br>contribution to the problem, only a<br>groan.	0
**10**	**1**	26/01/1941	28/03/1941	61	Dear Enid,<br>Vita is coming here on the 18th. I<br>wonder if you would come over to<br>lunch on the 19th (a Wednesday) at<br>one? It would be a great pleasure if you<br>would. She says you have a phaeton<br>and a hunter that jumps gates. We are<br>down here, bombed out of London.<br>Vita is lecturing the Womens<br>Institute, so I promised her I would try and<br>give her a treat next day. Yrs<br>Virginia Woolf	0

**11**	**1**	01/02/1941	28/03/1941	55	I have written you ever so many beautiful<br>letters, cigarette letters, you know the<br>kind, when ones devotion to Ethel rises<br>like a silver smoke, too fine for words.<br>These are the letters I write you, about<br>3 on a wet windy morning. Unlike<br>Margot, I dont keep a pencil at my<br>head and I forget where we left off,<br>you were going into the snow in snow<br>boots. You had seduced the wife of the<br>woodcutter and then? I have a far away lover, to match your translator, a<br>doctor, a cousin, a Wilberforce, who<br>lives at Brighton and has, by a miracle, heard of you. If I were in London, I<br>would ask you to meet. She has a herd of<br>Jersey cows and sends me a pot of<br>cream weekly. Oh theres Margot, I<br>cant fathom her, I get now almost<br>daily a letter written in bed at 3 am in<br>the Savoy. Why at this last lap of time<br>should she fabricate an entirely<br>imaginary passion for me, who am<br>utterly incongruous You and Frances<br>Horner she says this morning are the<br>only women I have ever loved. The rest<br>of womenkind, as I can well imagine,<br>seeing her clothes, she hates, Yet she assures me she never bedded with a<br>lover. And why assure me of anything?<br>Is it that at the end of life she must<br>somehow still collect some mirror? and<br>I, being unused, still reflect whats no	1
**12**	**1**	02/02/1941	28/03/1941	54	Re article in Sunday Times<br>No, no, no, my dear Desmond, I really<br>must protest. I never sat on top of a<br>tower! Compare my wretched little<br>150 education with yours, with<br>Lytton, with Leonard. Did Eton and<br>Cambridge make no difference to you?<br>Could the Hawk have been so affable<br>and so hawklike without it? Would<br>Lytton have written just as well if he would spent his youth, as I did mine,<br>mooning among books in a library? I assure you,<br>my tower was a mere toadstool, about<br>six inches high. And when you say She<br>herself as a writer owes everything to<br>having seen the world from a tower<br>which did not lean you make me<br>gnash my teeth. If you knew my<br>inadequacy; what shifts and squeaks<br>I am put to every time I dip my pen! Of<br>course I am not on the ground with the<br>WEA but I am about four thousand five<br>hundred and fifty pounds nearer them<br>than you are. So I am right to say we<br>when I talk to them; just as I am right to<br>say they when I look up, as I do with<br>constant envy and admiration, at you. This is the brief residue of a three<br>hours argument with Leonard. So I<br>thought I would pop my conclusion in an<br>envelope and send it you.<br>No chance I suppose of another week	1

**13**	**1**	02/02/1941	28/03/1941	54	Dear Enid,<br>What a muddle! Vita said how much<br>she would like to meet you. I said, Then I will<br>ask her over. Vita said, Perfect. Not a<br>sign that she had invited herself to you.<br>Perhaps we would better leave it as it is<br>if you will be so angelic as to come here. I<br>know its asking a lot. And its not true<br>that I hate leaving my house, not at all.<br>But this we can discuss when you come. So we expect you at One on the<br>19th. If you should ever see Maurice<br>Baring, could you convey my respectful<br>affection? Why are you North End House in<br>the Telephone; and Elms on your<br>paper? Yr<br>Virginia Woolf	1
**14**	**1**	03/02/1941	28/03/1941	53	Dear Sir,<br>I have to thank you for your letter of<br>31 January, but there has been no<br>muddle or misunderstanding on my<br>side. I have had no cable, letter, or any<br>other communication either from Mr<br>Davis or Miss McFadden. I had a letter<br>from your office asking me for a story,<br>saying that the American office had cabled to you. As I was engaged on a<br>book at the time. I was not anxious to<br>do it immediately and I replied that I<br>had nothing by me, but had some<br>stories roughly sketched out in my<br>London house and would get them and<br>look at them when I was next in town. I<br>also enquired whether this was a<br>commission, as I was not prepared to<br>write a story except it was<br>commissioned. I then received letters<br>from your office pressing me to get the<br>stories from my London house so that<br>you could send one on the Clipper [air<br>mail] to the U.S.A. as that was the<br>repeated cabled request from your<br>American office and informing me that it would definitely be a commission<br>from America. See your letters of<br>October 14, 20, and 25. I do not see payment will be made for the story<br>commissioned by you which will bring<br>in foreign exchange as effectively as if it<br>had been printed and after that I will	1

**15**	**1**	04/02/1941	28/03/1941	52	Oh dearest Vita, what an overflowing<br>Cornucopia you are! How you pet<br>pamper and spoil me! Nothing could<br>have come more pat than your pat. I would<br>shaken a bottle of milk for an hour; at<br>last a yellow lump appeared: I put it on<br>the kitchen table. The cat ate it. So<br>when the post came, it was like the<br>voice of God in answer to our prayers.<br>What did Staples [Nicolsons cook] say<br>when she found it gone? Yes, I am a butter maker now, and it takes the devil<br>of a time. Also: what am I wearing at this<br>moment? Jacobs Ram. Louie made me<br>a thick warm jersey. Its saved my life I<br>live in it. And its a lovely colour. The<br>whole county envies me. Dear me, how<br>you rain blessings. Enid, by the way,<br>writes (perhaps slightly aggrieved) that<br>you said you would lunch with her. So will I<br>come too? I have said no. She must come<br>here; and she will. Yes, we will get the<br>Lanthorn [for Vitas lecture].<br>I am going to London tomorrow to<br>walk among the ruins. Did I tell you all<br>my books are to bits? So, if you have<br>Lady Ann Clifford or any other Elizabethan biographer, dear me, I am<br>asking another favour; but could you<br>bring them?<br>Its the very devil writing when every<br>book lies at the bottom of a vast hole up	1
**16**	**1**	08/02/1941	28/03/1941	48	Dearest Ethel, of course I minded your<br>distemper with me, but of course I put<br>it down to misery. And now your card<br>makes all safe and sound again: I only<br>scrawl this by way of a hug, which<br>indeed I would like to give you, loving your<br>warm heart. Leonard says, he has not<br>read Curry: he says he doesnt believe in<br>Federal Union now. He says he has given<br>all his views on that in his 2/6 book.<br>War for Peace. If you would like to read it, I think its very masterly,I will send<br>it.<br>Lord! What a horror about the<br>insects WC on Pans nose! Enough to<br>make anyone tart even with a woman<br>who loves you with every fibre of what<br>is in fact a damned good heart. I was<br>out of spirits I admit all day after your<br>letter. And what do you mean, I ask, by<br>your you might do a tiny bit of direct<br>work for your living Ought I, do you<br>mean, not to write articles? I agree. The<br>Thrale is nonsense, too, as I have to<br>press it all into 6 pages, damn these<br>editors! Off to Letchworth and Cambridge<br>next week to see the Press. Oh Lord,<br>what an effort moving in this weather and these conditions. Our line was<br>blown up on Wednesday and it took 3<br>hours to get back from London Yr<br>inarticulate but adoring.	1

**17**	**1**	10/02/1941	28/03/1941	46	Mary! Weasel! I have been trying to write<br>this letter in hand writing, but my hand<br>is like the cramped claw of an aged<br>fowl: so I turn to the type. Please<br>forgive. An odious habit.<br>No, I dont think I can write about<br>The English and French. I have sat staring<br>at it, but its too vast, too oleaginous.<br>Nothing whatever happens. You see<br>one has to approach ones subject with a little thrill, as if you were opposite<br>and I suddenly gave you a rose. So I have told<br>your old Bugger that I wont do that;<br>but if they allow an English subject, a<br>writer, then I will do my best. If one could<br>amuse the French, poor people, I<br>would. Salute the man in your<br>basement from me. I read his story in<br>the paper. And to think of you on a<br>battleship! As you say, wheres one to begin?<br>Yes, Leonard has got a purple hyacinth.<br>And the flood has gone. Then the snow<br>came, and I made green holes in the<br>grass every time I came out here to my<br>Lodge. Now the snow has gone. Life is<br>rapid but eventless. We take tea at Charleston: Clive is digging a trench;<br>Nessa feeding fowls; Duncan painting<br>Christ; Quentin driving a tractor, all as<br>it was in 1917. Tomorrow we go for a<br>jaunt, to Cambridge, taking the Press at<br>Letchworth in our way. And we shall	1
**18**	**1**	17/02/1941	28/03/1941	39	Dear Pippa,<br>I found your letter here, and am very<br>grateful. It was angelic of you to go into<br>the matter. Alas, Leonard has plumped<br>in favour of it, so I have accepted, I<br>neednt say how reluctantly. But I<br>suppose it dont much matter. I can<br>always resign in a huff at a word from<br>you. What a bore it was that you were not<br>at Cambridge! We had a long gossip with Pernel, and felt as if we had had a<br>hot bath. it was so clean warm and<br>civilised. But we couldnt stay this time.<br>Next time we must see you, which<br>would rejuvenate us. I suppose you<br>cant come south? No. How d-d this<br>war is! Yr Virginia	1
**19**	**1**	19/02/1941	28/03/1941	37	Dearest Dadie,<br>I had meant to thank you before for<br>the extraordinarily happy evening you<br>gave us. It was only that coming back<br>here to find Elizabeth [Bowen], Vita<br>and Lady Jones I could not seize a pen. It<br>remains like an oasis, last Wednesday,<br>not a mirage, in the desert.<br>I have ordered the books you bade me,<br>and thank you for everything and we<br>both send our love.	1

**20**	**1**	23/02/1941	28/03/1941	33	Dear Octavia,<br>You have reduced me not to silence<br>quite, but to a kind of splutter, I mean,<br>the cream; the cheese: the milk. I dont<br>see how to begin: and twice a week the<br>debt mounts. And you dont come here<br>so that I could speak by word of mouth.<br>Dear, dear. I am dumb. But can just say<br>we had a magnificent feast of cheese<br>last night: not had one since September:<br>and seldom any so suave and sweet and yet sour. No I did not add sugar.<br>For there was a natural sweetsour in it that<br>was best unmixed. We too have been turmoiled, not, I<br>expect, for any such good purpose as<br>you. Only going to Cambridge, to<br>Letchworth, and somehow having a run<br>of visitors in the house. Should you be able to come over,<br>please suggest it. And, have I the face<br>to add, does your cook find any Seville<br>oranges in the Brighton shops? None<br>here. I daresay none anywhere. But I only want to suggest a visit<br>some time.	1
**21**	**1**	01/03/1941	28/03/1941	27	Do you know, I have written you three<br>separate letters, and torn each of them<br>up? This is a fact. Partly, they were<br>dull: partly something always<br>interrupted. Ever since we came back<br>from Cambridge, 30 hours in train<br>journeys: 6 on hotel bills, all for<br>Leonard to spend two hours at<br>Letchworth, I have been in a fret. People<br>kept turning up. Oh yes, there was<br>Vita, and Enid Jones to lunch. You know, if ones only got a half daily maid<br>its difficult, getting food together: and<br>the wine had run out; and the duck<br>was all strings and blue sinews.<br>However Enid was as dapper as a dab<br>chick. A brick I think would be the<br>proper word, something a bit gritty<br>and granular; but hard to the foot. Of<br>course she, an old love I fancy,<br>wanted to be alone with Vita; and there<br>I was; and it was pelting wet, the cat<br>had scratched a hole in the chair cover,<br>and a visiting dog had lifted his leg<br>against the table, In short there was an<br>atmosphere of the sordid and squalid. I<br>gave her your message, and she<br>kindled, and said I was to tell you, it was when I got to this point in the<br>other letters that I stopped: it bores me<br>so repeating messages; she said Maurice<br>[Baring] is much better on his Scotch	1

**22**	**1**	04/03/1941	28/03/1941	24	Oh dearest Creature, now you have<br>topped the whole hill of your<br>benefactions with a firelighter. Po:<br>butter: wool: books: firelighter on top.<br>There you must stop. You cant add<br>anything to fire. You see the poetic<br>fitness of ending there. What a<br>magnificent conception of life you have<br>O damn the law. Leonard says we<br>cant use your petrol. Another gift. But<br>it appears there is a Bus. Couldnt we arrange that? I suppose your orchard<br>is beginning to dapple as it did the day I<br>came there. One of the sights I shall see<br>on my death bed. I suppose you havent any Hay to sell?<br>Octavia Wilberforces cows at Henfield,<br>which give us butter, are starving. So I<br>said I would ask. Silence means no.<br>I have been ranging the country this<br>afternoon, asking for hay. Not a blade<br>to be had. No, Ive not read Enids play [Lottie<br>Dundas]. Would she lend it me? Is it a<br>masterpiece? or merely a moneymaker?<br>The Anrep flat fell through, damn it.<br>Oh to think I shall never sit in the cold again. Aint this a pretty pattern for<br>a letter?	1
**23**	**1**	04/03/1941	28/03/1941	24	Dear Octavia, Excuse this typing; which is my<br>method of trying to be exact and<br>practical. Its about the flat. I find that<br>Helen Anrep wants two bed rooms; if<br>possible three; kitchen and bath room;<br>and sitting room. She cant afford more<br>than 50. I imagine this puts it out of<br>the question. Should you hear of<br>anything, then tell me; but on no account trouble. Also, as far as I can<br>make out, the old part of Brighton is<br>what she likes. Whether this is dearer,<br>or cheaper, I dont know. There! That<br>sounds very pat. And now of course typing dont suit<br>any other kind of letter. So I wont begin<br>on the question of my living portrait.<br>All I say is, I see that no one can be<br>asked to sit. Why should they?<br>Wouldnt it be a kind of torture? It was<br>only a wild flitting dream. I will try to<br>write to Miss Robins. No, no, no, I<br>cannot write on a typewriter; and so<br>must give over and say once more, what<br>a damned generous woman you are.<br>Have you any use for bottled gooseberries? Many pots here if you<br>would take them.<br>[handwritten:] All the same, I add in handwriting I<br>think youre very paintable, as the<br>painters say. Now I wonder why?<br>Something that composes well.<br>Perhaps reticence and power combined:	1

**24**	**1**	08/03/1941	28/03/1941	20	My dear Tom, With Mr Eliots compliments, how<br>can I answer that very brief<br>communication? Only I suppose by<br>returning Mrs Woolfs comps. As you<br>know, profound inhibitions prevent me<br>from saying anything about the poem.<br>Happily, they dont prevent me from<br>thanking you very profoundly for<br>remembering Mrs Woolf. I have had it on the tip of my<br>typewriter these many weeks to suggest<br>that time is passing; and it would be a<br>great pleasure to fix a point and see<br>you. I suggest 5th of April week end. I<br>do so very tentatively, because I know<br>havent we just been to Cambridge<br>and back, the horror of trains. But our<br>line is still better than some. Then<br>theres the lack of civility here. Its a<br>dripping day; the water has come<br>through the kitchen ceiling.<br>All the same, if you would venture,<br>theres nothing we should like better.<br>Or the middle of the week, if thats<br>more to your liking. So much water has flowed under the bridge that I feel at<br>sea; and so conclude.	1
**25**	**1**	10/03/1941	28/03/1941	18	Yes, yes yes, of course I agree with you.<br>This refers to Mr. Currys book, which<br>I have just read. But then of course I am<br>not a politician, and so take one leap to<br>the desirable lands. Leonards view would be, I<br>think, that ones got to plod along the<br>road, indeed to make it, before one gets<br>there. But Lord! what a relief to have a<br>vision! and I am glad youre beating up<br>an audience in Woking:<br>What I really write to say though is, how damnable these separations are!<br>Letters, no letters; then letters again.<br>Shall I come down for a night? I could<br>now, on a Wednesday, in April, go<br>back early Thursday.<br>But I daresay you cant manage.<br>Anyhow youre off to Winnie. But when<br>youre back lets try to bridge this<br>solitude. I would bring my rations. I am<br>in the dither of trying to contrive spring<br>cleaning. Oh our carpets, I spent 2<br>hours carpet beating, and still the flakes<br>of our bombed ceiling flock, and drown<br>the books just dusted. I had no notion,<br>having always a servant, of the horror<br>of dirt. No: politics at the moment seem more pressing than autobiography.<br>We<br>have the drone of raiders every night,<br>and the village is now fire spotting,<br>chiefly incendiaries away over the hill.	1

**26**	**1**	13/03/1941	28/03/1941	15	Dear Miss Robins, I was very sorry to hear from Octavia<br>[Wilberforce] that you had had an<br>accident. Selfishly, I am afraid it may<br>interfere with the book that I am looking<br>forward to. But I remember a saying of<br>Henry James, all experiences are of use<br>to a writer. I think he was talking about<br>a nervous breakdown. So may it be<br>worth a broken bone.<br>I now go on to say that I have been cycling into Lewes, not a very<br>interesting remark, save that it connects<br>with Octavia. Has she told you, I<br>wonder, no, I dont suppose she has,<br>of her amazing bi-weekly bounty,<br>cream, milk, sometimes a cheese? Thats<br>what I have been fetching. You cant think<br>how it brightens our weekly bill of fare.<br>Also, to fetch her empty basket, she<br>sometimes comes over, and this has<br>been, is, and will be, I hope, a great<br>treat. Is it our drop of blood in<br>common? Anyhow we sit over the fire,<br>as if we had known each other in the<br>woods at Lavington. Its odd how our<br>lives have run just not meeting but<br>through the same country. Thats the sort of woman I most admire, the<br>reticence, the quiet, the power. Here I<br>can imagine her look of enquiry. why?<br>Well its difficult to say why. Its the<br>variety and the calm partly. As you can	1
**27**	**1**	16/03/1941	28/03/1941	12	Dear Ruth,<br>Yes, I was very pleased with the<br>reviews of Roger, especially the ones<br>that abused him, for it shows how<br>much bite he still has. But the one I<br>liked best was J. T. Sheppards in the<br>Cambridge Review, for that said that<br>Roger Fry was there and not V.W.,<br>which was what I wanted.<br>You have been so good, taking an<br>interest in it, I mean considering me, the author, thank you so much. It still<br>goes on selling; but owing to an idiotic<br>blunder, the Press gave me no time to<br>correct misprints and mistakes. I must<br>apologise; and will put them in, should<br>there be another edition.<br>We are being a good deal bombed at<br>the moment. Rows of incendiaries fell<br>on the farm two nights ago and burnt<br>haystacks, but so far no one has been<br>hurt. I suppose Thorpeness [Suffolk] is<br>about as much of a target as we are.<br>Hoping we shall both escape, and<br>meet some day.<br>Your affectionate	1

**28**	**1**	18/03/1941	28/03/1941	10	Dearest,<br>I feel certain that I am going mad<br>again: I feel we cant go through mother<br>of those terrible times. And I shant<br>recover this time. I begin to hear voices,<br>and cant concentrate. So I am doing<br>what seems the best thing to do. You<br>have given me the greatest possible<br>happiness. You have been in every way<br>all that anyone could be. I dont think<br>two people could have been happier till this terrible disease came. I cant<br>fight it any longer, I know that I am spoiling<br>your life, that without me you could<br>work. And you will I know. You see I<br>cant even write this properly. I cant<br>read. What I want to say is that I owe<br>all the happiness of my life to you. You<br>have been entirely patient with me and<br>incredibly good. I want to say that,<br>everybody knows it. If anybody could<br>have saved me it would have been you.<br>Everything has gone from me but the<br>certainty of your goodness. I cant go on<br>spoiling your life any longer.<br>I dont think two people could have<br>been happier than we have been.	1
**29**	**1**	20/03/1941	28/03/1941	8	Dear John,<br>I have just read my so called novel<br>[Between the Acts] over; and I really<br>dont think it does. Its much too slight<br>and sketchy. Leonard doesnt agree. So<br>we have decided to ask you if you would mind<br>reading it and give your casting vote?<br>Meanwhile dont take any steps.<br>I am sorry to trouble you, but I feel<br>fairly certain it would be a mistake from all points of view to publish it.<br>[handwritten:] But as we both differ<br>about this, your opinion would be a<br>great help. Yours<br>I hope youre sending the manuscripts,<br>I should like to do them.	1

**30**	**1**	21/03/1941	28/03/1941	7	My dear Nelly,<br>To begin with business, Leonard<br>review came out in the N.S. on the 15th<br>February. I am glad that business has caused you<br>to break ten years of silence. When<br>petrol is scarce, one must have recourse<br>to ink. I wish we could come over [to<br>Gale, Chelwood Gate], but the car<br>only gets as far as Lewes once a week.<br>I am so sorry that Lord Robert never sent me his book. I should have liked to<br>read it in my own copy. I read it in<br>Leonards, and wished for more. There<br>wasnt, even for me, a non-politician, a<br>word too much about the League, but I<br>wished for more about Grove End Road<br>[London] and the room looking onto<br>the garden, and you. Is this vulgar? I<br>suppose so. I am greatly flattered that his<br>old opinion of me a wrinkled hag<br>has another side to its face. Often when<br>I turn over my old scrap book at night I<br>pause at the Gale page.<br>I am glad you liked Leonards book<br>[The War for Peace], and gave it to a<br>sceptic. It seemed to me the only kind<br>of thing worth writing now. Do you find you can read the novelists? I cant.<br>Still, I agree that this war is better than<br>last, and ever so much better than the<br>last 5 years of peace. We have been<br>bombed out of London and live entirely	1
**31**	**1**	21/03/1941	28/03/1941	7	Dear Susie,<br>How very nice of you to write! I<br>hadnt liked to write to you, but I did<br>want to hear about you. I know how<br>difficult it must be, making a fresh start.<br>But you have a great deal more than<br>most of us to look forward to. Elizabeth<br>[Bowen] told me too of all your war<br>activities. I expect that is a help too. I<br>wanted also to write to you when your<br>mother died. The memory of her goes back so far in my life, I remember<br>dining out for the first time in your<br>house, after your fathers death, and I<br>remember him too coming to see us,<br>and admiring him, as a child. Is not one<br>of your sons like him? I saw a<br>photograph, and thought so.<br>We have been completely bombed<br>out of London, and lead a rather<br>vegetable existence here, surrounded by<br>the melancholy relics of our half<br>destroyed furniture. All this afternoon<br>I have been trying to arrange some of my<br>fathers old books.<br>Only now and then do I come to<br>London at present. May I let you know<br>a date later in case we could meet? I am always rather rushed, thats the<br>worst of it, as we have nowhere to stay in town.<br>Yes, I think thats a very good idea,<br>writing letters from the country to	1

**32**	**1**	22/03/1941	28/03/1941	6	Look at this letter [lost], sent to the<br>New Statesman, addressed to Miss<br>Virginia Woolf. What a queer<br>thought transference! No, I am not you.<br>No, I dont keep budgerigars.<br>Louies survive: and she feeds them<br>on scraps. I suppose they are lower<br>class, humble, birds. If we come over<br>[to Sissinghurst], may I bring her a pair<br>if any survive? Do they die all in an<br>instant? When shall we come? Lord knows.	1
**33**	**1**	23/03/1941	28/03/1941	5	Dear John,<br>I have gone through these MSS as far<br>as I can. But my head is very stupid at<br>the moment.<br>The only ones that are I think worth<br>considering are: Mr Robinsons poems;<br>and Mr Urquarts story. Both have<br>distinct merit, I think; though both are<br>border line cases. Yours ever	1
**34**	**1**	23/03/1941	28/03/1941	5	Dearest,<br>You cant think how I loved your<br>letter. But I feel that I have gone too for<br>this time to come back again. I am<br>certain now that I am going mad again.<br>It is just as it was the first time, I am<br>always hearing voices, and I know I<br>shant get over it now.<br>All I want to say is that Leonard has<br>been so astonishingly good, every day,<br>always; I cant imagine that anyone could have done more for me than he<br>has. We have been perfectly happy until<br>the last few weeks, when this horror<br>began. Will you assure him of this? I<br>feel he has so much to do that he will<br>go on, better without me, and you will<br>help him. I can hardly think clearly any more.<br>If I could I would tell you what you<br>and the children have meant to me. I<br>think you know. I have fought against it, but I cant<br>any longer.	1

**35**	**1**	27/03/1941	28/03/1941	1	Dear John,<br>I had decided, before your letter came,<br>that I cant publish that novel as it<br>stands, its too silly and trivial.<br>What I will do is to revise it, and see<br>if I can pull it together and so publish it<br>in the autumn. If published as it is, it<br>would certainly mean a financial loss;<br>which we dont want. I am sure I am<br>right about this. I neednt say how sorry I am to have<br>troubled you. The fact is it was written<br>in the intervals of doing Roger with my<br>brain half asleep. And I didnt realise<br>how bad it was till I read it over.<br>Please forgive me, and believe I am<br>only doing what is best.<br>I am sending back the MSS [for Folios<br>of New Writing] with my notes.<br>Again, I apologise profoundly.	1
**36**	**1**	28/03/1941	28/03/1941	0	Dearest,<br>I want to tell you that you have given<br>me complete happiness. No one could<br>have done more than you have done.<br>Please believe that.<br>But I know that I shall never get over<br>this: and I am wasting your life. It is<br>this madness. Nothing anyone says can<br>persuade me. You can work, and you<br>will be much better without me. You<br>see I cant write this even, which shows I am right. All I want to say is that<br>until this disease came on we were perfectly<br>happy. It was all due to you. No one<br>could have been so good as you have<br>been, from the very first day till now.<br>Everyone knows that.<br>You will find Roger's letters to the<br>Maurons in the writing table drawer in<br>the Lodge. Will you destroy all my<br>papers.	1

**37**	**0**	01/01/1941	28/03/1941	86	On Sunday night, as I was reading<br>about the great fire, in a very accurate<br>detailed book, London was burning. 8<br>of my city churches destroyed, & the<br>Guildhall. This belongs to last year.<br>This first day of the new year has a slice<br>of a wind like a circular saw. Leslie H.<br>came to lunch; said um-um so often I<br>nearly goggled; he was discussing the foundations of communism, having<br>come chiefly to pick Leonards brain. Gossip in<br>between; then old Octavia came, with<br>her market womans basket. Great white<br>bottles of milk & cream. L. looking at<br>the comet. Rather a strong moon, & so<br>cant identify the constellation. Mrs<br>Coleridge Taylor tapped at the door;<br>about a concert. And now its close on<br>cooking time. This book was salvaged<br>from 37: I brought it down from the<br>shop, with a handful of Elizabethans for<br>my book, now called Turning a Page.<br>A psychologist would see that the above<br>was written with someone, & a dog, in<br>the room. To add in private: I think I<br>will be less verbose here perhaps but what does it matter, writing too many<br>pages. No printer to consider, no public.	0
**38**	**0**	09/01/1941	28/03/1941	78	A blank. All frost. Still frost. Burning<br>white. Burning blue. The elms red. I<br>did not mean to describe, once more,<br>the downs in snow; but it came. And I<br>cant help even now turning to look at<br>Asheham down, red, purple, dove blue<br>grey, with the cross so melodramatically<br>against it. What is the phrase I always<br>remember or forget. Look your last on<br>all things lovely.<br>Yesterday Mrs Dedman was buried<br>upside down. A mishap. Such a heavy<br>woman, as Louie put it, feasting spontaneously upon the grave. Today<br>she buries the Aunt whose husband<br>saw the vision at Seaford. Their house<br>was bombed by the bomb we heard<br>early one morning last week. And L. is<br>lecturing & arranging the room. Are<br>these the things that are interesting?<br>that recall; that say Stop you are so fair?<br>Well, all life is so fair, at my age. I<br>mean, without much more of it I<br>suppose to follow. And to other side of<br>the hill there will be no rosy blue red<br>snow. I am copying P.H. I am<br>economising. I am to spend nothing.<br>One day, 11 years ago I spent 2.2 on<br>glass jars. That was the loosening of the<br>purse & I said it was difficult. Is it difficult now to string tight? The great<br>change is not that but the change to the	0

**39**	**0**	15/01/1941	28/03/1941	72	Parsimony may be the end of this<br>book. Also shame at my own verbosity,<br>which comes over me when I see the 20<br>it is books shuffled together in my<br>room. Who am I ashamed of? Myself<br>reading them. I answered David Cecils silly sneer at<br>Lytton & Mrs Woolf, withdrawing from<br>life to cultivate their art in quiet. The<br>little man I suppose justifies himself by<br>sneering at us. Then Joyce is dead<br>Joyce about a fortnight younger than I<br>am. I remember Miss Weaver, in wool<br>gloves, bringing Ulysses in type script to<br>our tea table at Hogarth House. Roger I<br>think sent her. Would we devote our<br>lives to printing it? The indecent pages<br>looked so incongruous: she was<br>spinsterly, buttoned up. And the pages<br>reeled with indecency. I put it in the<br>drawer of the inlaid cabinet. One day<br>Katherine Mansfield came, & I had it<br>out. She began to read, ridiculing: then suddenly said, But theres some thing<br>in this: a scene that should figure I<br>suppose in the history of literature. He<br>was about the place, but I never saw<br>him. Then I remember Tom in<br>Ottolines room at Garsington saying<br>it was published then how could<br>anyone write again after achieving the<br>immense prodigy of the last chapter?	0
**40**	**0**	20/01/1941	28/03/1941	67	I will be curt, compressed. A mood<br>like another. Back from a damp, perhaps rather strained, visit to<br>Charleston. Nessa & Quentin; Adrian<br>has almost died of pneumonia. Nessa<br>apprehensive, on guard, when I spoke<br>of Angelicas dirt. Search for<br>epidiascope in Lewes. Fruitless. Lecture<br>tomorrow. 5 small trout for lunch.<br>Octavias cream. Talk of soup making.<br>Reading Gide. La Porte Etroite [1909]<br>feeble, slaty, sentimental.<br>Visit from Oliver Strachey. All stocky<br>gloom. Flogged my brain for topics.<br>Lighted on the war. Civilisation over for<br>500 years. And my life is at an end.<br>Enter two breezy brisk colleagues. He<br>shares a sitting room. I lost several<br>pages of PH. I say to Nessa, Do you find painting gets slower? Yes. One can<br>do more. And money? Never think of<br>it. And Helen? She does nothing. I like<br>being alone. How can one do nothing?<br>Duncan coming & Clive. All the same<br>MH is somehow cheerful. Q. has an<br>offer of a draughtsman job at Dorking.<br>Better than farm work. The Girls school<br>at Lewes is behind Ann of Cleves<br>House, a large, tiled, swept, clamorous<br>place. The headmistress large & tight,<br>practical. No one knows we exist she	0

**41**	**0**	26/01/1941	28/03/1941	61	A battle against depression, rejection<br>(by Harpers of my story & Ellen Terry)<br>routed today (I hope) by clearing out<br>kitchen; by sending the article (a lame<br>one) to N.S.: & by breaking into PH 2<br>days, I think, of memoir writing.<br>This trough of despair shall not, I<br>swear, engulf me. The solitude is great.<br>Rodmell life is very small beer. The<br>house is damp. The house is untidy.<br>But there is no alternative. Also days<br>will lengthen. What I need is the old<br>spurt. Your true life, like mine, is in<br>ideas Desmond said to me once. But<br>one must remember one cant pump ideas. I begin to dislike introspection.<br>Sleep & slackness; musing; reading;<br>cooking; cycling; oh & a good hard<br>rather rocky book viz: Herbert Fisher.<br>This is my prescription. We are going to<br>Cambridge for two days. I find myself<br>totting up my friends lives: Helen at<br>Alciston without water; Adrian &<br>Karin; Oliver at Bedford, & adding up<br>rather a higher total of happiness.<br>There is a lull in the war. 6 nights<br>without raids. But Garvin says the<br>greatest struggle is about to come, say<br>in 3 weeks, & every man, woman dog<br>cat even weevil must girt their arms,<br>their faith, & so on.	0
**42**	**0**	07/02/1941	28/03/1941	49	Why was I depressed? I cannot<br>remember.<br>We have been to Charlie Chaplin.<br>Like the milk girl we found it boring. I<br>have been writing with some glow. Mrs<br>Thrale is to be done before we go to<br>Cambridge. A week of broken water<br>impends. Cambridge; then Elizabeth<br>Bowen; then Vita & Enid Jones. Helen has repaid me 25. Do I like her better<br>for it? I think so. The snow came back.<br>Marshes in the thaw a swamp. We were<br>in London [on 5 February], & had to<br>come home, owing to a bomb, by<br>Dorking; the car was locked at Martins<br>[garage]; we dined at the White Hart<br>poor soup & oxtail; London streets are<br>very empty Oxford Street a wide grey<br>ribbon. My red purse bag stolen & L.<br>gave me another. At Charleston Clive<br>was stockish, like a Bell. I said What a<br>risk Nessa ran marrying him! The<br>Sitwells are proving their existence as<br>poets in the Law Courts. This is<br>despicable but delightful. And what<br>else? [Ellen] Terry appears today in the N.S. The Italians are flying. The 3rd<br>week in March is fixed for invasion.<br>Now black out, & perhaps write to<br>Mary. No I think read. what? I must<br>tune up for my Elizabethans.	1

**43**	**0**	16/02/1941	28/03/1941	40	In the wild grey water after last<br>weeks turmoil. I liked the dinner with<br>Dadie best. All very lit up &<br>confidential. I liked the soft grey night<br>at Newnham. We found Pernel in her<br>high ceremonial room, all polished &<br>spectatorial. She was in soft reds &<br>blacks. We sat by a bright fire. Curious<br>flitting talk. She leaves next year.<br>Then Letchworth, the slaves chained to their typewriters, & their<br>drawn set faces, & the machines the<br>incessant more & more competent<br>machines, folding, pressing, glueing &<br>issuing perfect books. They can stamp<br>cloth to imitate leather. Our Press is up<br>in a glass case. No country to look at.<br>Very long train journeys. Food skimpy.<br>No butter, no jam. Old couples<br>hoarding marmalade & grape nuts on<br>their tables. Conversation half<br>whispered round the lounge fire. Eth<br>Bowen arrived two hours after we got<br>back, & went yesterday; & tomorrow<br>Vita; then Enid; then perhaps I shall reenter<br>one of my higher lives. But not<br>yet.	1
**44**	**0**	26/02/1941	28/03/1941	30	My higher life is almost entirely the<br>Elizabethan play. Finished Pointz Hall,<br>the Pageant: the Play finally Between<br>the Acts this morning. Flora & Molly<br>have just gone; leaving me to ask this<br>bitter bright spring day, why they came?<br>Yesterday in the ladies lavatory at the<br>Sussex Grill at Brighton I heard:<br>She is a little simpering thing. I dont<br>like her. But then he never did care for<br>big women. (So to Bert) His eyes are so<br>blue. Like blue pools. So is Gerts. They<br>have the same eyes, only her teeth part<br>a little. He has wonderful white teeth.<br>He always had. Its fun having the boys If he dont look out he will be court<br>martialed.<br>They were powdering & painting,<br>these common little tarts, while I sat,<br>behind a thin door, painting as quietly as<br>I could.<br>Then at Fullers. A fat, smart woman,<br>in red hunting cap, pearls, check skirt,<br>consuming rich cakes. Her shabby<br>dependant also stuffing. Hudsons van<br>unloading biscuits opposite. The fat<br>woman had a louche large white muffin<br>face. To other was slightly grilled. They<br>ate & ate. Talked about Mary. But if<br>she is very ill, you will have to go to her.<br>Youre the only one But why should	1

**45**	**0**	08/03/1941	28/03/1941	20	Just back from Leonards speech at Brighton. Like a foreign town: the first spring day. Women sitting on seats. A pretty hat in a teashop how fashion revives the eye! And the shell encrusted old women, rouged, decked, cad verous at the tea shop. The waitress in checked cotton.<br>No: I intend no introspection. I mark Henry Jamess sentence: Observe perpetually. Observe the oncome of age. Observe greed. Observe my own despondency. By that means it becomes serviceable. Or so I hope. I insist upon spending this time to the best advantage. I will go down with my colours flying. This I see verges on introspection; but does not quite fall in.<br>Suppose, I bought a ticket at the Museum; biked in daily & read history.<br>Suppose I selected one dominant figure in every age & wrote round & about.<br>Occupation is essential. And now with some pleasure I find that its seven; & must cook dinner. Haddock & sausage meat. I think it is true that one gains a certain hold on sausage & haddock by writing them down.<br>Last night I analysed to Leonard, my London Library complex. That sudden terror has vanished; now I am plucked at by the H. Hamilton lunch that I	1
**46**	**0**	24/03/1941	28/03/1941	4	She had a face nose like the Duke of Wellington & great horse teeth & cold prominent eyes. When we came in she was sitting perched on a 3 cornered chair with knitting in her hands. An arrow fastened her collar. And before 5 minutes had passed she had told us that two of her sons had been killed in the war. This, one felt, was to her credit. She taught dressmaking.<br>Everything in the room was red brown & glossy. Sitting there I tried to coin a few compliments. But they perished in the icy sea between us. And then there was nothing.<br>A curious sea side feeling in the air today. It reminds me of lodgings on a parade at Easter. Everyone leaning against the wind, nipped & silenced. All pulp removed. This windy corner. And Nessa is at Brighton, & I am imagining how it wd be if we could infuse souls.<br>Octavias story. Could I englobe it somehow? English youth in 1900.<br>Two long letters from Shena & O. I cant tackle them, yet enjoy having them.<br>Leonard is doing the rhododendrons …	1

**47**	**0**	03/01/1940	28/03/1941	450	Monks House, Rodmell<br>This very large sheet which I bought<br>at Baxters [Lewes stationers] two days<br>ago begins a new year, on a new<br>system. Evening over the fire writing,<br>instead of end of the morning<br>scrambling. Thus I hope to write a<br>better hand, &, if it werent that Ive<br>just heated my head over Roger, the PIP [Post-Impressionists] (a bad<br>chapter) more solidly. For unless I can<br>put a little weight into this book, it will<br>have no interest, even for an old<br>woman, turning the pages. I have just<br>put down Mills autobiography, after<br>copying certain sentences in the volume<br>I call, deceptively, the Albatross. We<br>have been out in Janices car, looking<br>for skating. Its a long bitter winter frost<br>I forget how many degrees of a night<br>I think 22 below freezing. Figure an<br>Italian sun yesterday; & hard white<br>snow; & the street like glass; the village<br>treat to Brighton; chains round the<br>wheels; the butcher saying he had had<br>enough of it, which, as he has to be in the shop cutting joints at 6, I can<br>follow.<br>I am oppressed & distracted with all<br>my ideas. All the little cuckoos shoving<br>the old bird Roger out of the nest. A<br>book on W [ome] n & peace & here is.	0
**48**	**0**	30/01/1940	28/03/1941	423	Unable to go to London because of<br>the worst of all frosts. A sudden return.<br>Everything glass glazed. Each blade is<br>coated, has a rim of pure glass. Walking<br>is like treading on stubble. The stiles &<br>gates have a shiny green varnish of ice.<br>Percy has to dig paths. Ink frozen. On<br>Sunday no cars cd. move. Nessa said<br>the Lewises had to give up. Thats the<br>last I heard from Charleston. On<br>Monday the electric light failed.<br>Cooked breakfast on dining room fire.<br>Came on at 12.30. Today all idea of<br>travel impossible. Trains hours late or<br>lost. No buses running. Walked to<br>Lewes & back. Met snow plough; 2 or 3<br>cars; no walkers. Lewes very empty. Home by the short cut; which was<br>painful. A great flight of wild geese.<br>The grass is brittle, all the twigs are<br>cased in clear brown cases, & look<br>thick, but slippery, crystallised, as if<br>they were twigs of fruit at dessert. Now<br>& then the wireless reports a ship sunk<br>in the North Sea. Almost out of meat,<br>but at last the Coop sent. Very still<br>tonight. More snow? No papers till the<br>afternoon.	0

**49**	**0**	09/02/1940	28/03/1941	413	For some reason hope has revived.<br>Now what served as bait? A letter from<br>Joe Ackerley approving my Corelli? Not<br>much. Tom dining with us? No. I think<br>it was largely reading Stephens<br>autobiography: to it gave me a pang of<br>envy, by its youth & its vigour, & some<br>good novelists touches I could pick holes<br>though. But its odd, reading that &<br>South Riding both mint new, give me a<br>fillip after all the evenings I grind at<br>Burke & Mill. A good thing to read ones contemporaries, even rapid<br>twinkling slice of life novels like poor<br>W. H. And then, I have polished off, to<br>the last gaiter button, the 3 d,<br>chapters for London on Monday; & got<br>my teeth I think firm into the last<br>Transformations; & though of course I<br>shall get the black shivers when I reread<br>let alone submit to Nessa & Margery, I<br>cant help thinking I have caught a good<br>deal of that iridescent man in my oh so<br>laborious butterfly net. I daresay I have<br>written every page, certainly the last<br>10 or 15 times over. And I dont think<br>I have killed: I think I have brisked. Hence<br>an evening glow Yet the wind cuts like<br>a scythe: the dining room carpet is turning to mould; & John Buchan has<br>fallen on his head & is, apparently,<br>dying. I have already composed a letter	0
**50**	**0**	24/03/1940	28/03/1941	369	A curious sub-life has set in, rather<br>spacious, rather leisured, & secluded &<br>content. Still sleep in Leonards room; then I<br>slowly bath & dress & sit in the sitting<br>room, quietly, entering Ms<br>corrections. Not much bothered really,<br>though I have so short a time. I cant help<br>thinking, in spite of Leonard, that its<br>interesting: & I can I think liven &<br>improve. And I am buoyed up by M. &<br>N. & D. And feel in one fortnight I<br>shall be quit (oh no, there will be proofs).<br>This is an egg shell life, so gingerly do I<br>step to avoid rousing my temp: which<br>was 99 pt 4 & is again a little up: but if I dont walk in the wind I think I can<br>refrigerate, & yet keep my brain calm<br>for the morning. Wobbly like one of the<br>spring lambs in my legs. Mabel<br>increases comfort 100 fold. And its<br>refreshing & rejuvenating to see the<br>gold thick clumps of crocuses, & the<br>unopened green daffodils, & to hear my<br>Asheham rooks dropping their husky<br>caws through the gummy air. Birds are<br>having a trial. Leonard works all the afternoon<br>in his blue shirt making the rock<br>garden. Old Botten is dying. Mabel &<br>Louie have gone to church at<br>Southease, & the Lambs bleat.<br>I am beginning Sense & Sensibility &	0

**51**	**0**	26/03/1940	28/03/1941	367	A curious letter from Hugh this<br>afternoon, part of which I will copy, for<br>I like reading old letters.<br>As to my book (I wrote about<br>Roman Fountain) of course I knew that<br>you would dislike some of it very much,<br>but hoped you wd like some which<br>apparently you do. MacCarthy speaks<br>to me as to a child, so does Harold N.<br>talking of my babyish love of my toys in the D.T. But do you care to hear the<br>truth? Half of me is very mature, half<br>has never grown up at all. I cant help<br>my excitement which irritates you all. I<br>never had anything when I was young<br>(cant read). As to my writing you & I<br>are the opposite ends of the bloody<br>stick? You are the supreme example of<br>the aesthetic-conscience there has<br>never been such another in English<br>fiction. But you dont write novels. What<br>you write needs a new name. I am the<br>true novelist a minor one but a true<br>one. I know a lot about the novel & a<br>lot about life seen from my very twisted<br>child-haunted angle. Had I been<br>normal I might have been a major novelist. As it is I am a Siamese twin.<br>No I cant be bothered to copy any<br>more. It rushes up into a Bengal light<br>spirit of self-glorification crocuses,<br>sausages, Harold & chess at the end,	0
**52**	**1**	07/05/1940	28/03/1941	325	Forgive me for my wire, and the Saturday? About a fortnight later? Well,<br>I know its my fault, but the Frys have<br>sent so many last minute corrections,<br>and it means much more re-writing<br>than they know. and I am so slow and<br>get so muddle headed.<br>Would you also tell Ben [Nicolson]<br>that I waited for him to ring me up<br>again (at 37) and hoped to arrange<br>something. I do want to see him. Whats<br>his address?<br>I will write and suggest a dinner next<br>time we are up.<br>Your wretched drudge, oh how I want to come on<br>Saturday!	0

**53**	**1**	08/05/1940	28/03/1941	324	Dear Mr Hart-Davis,<br>Many thanks for telling me about the<br>book, which I shall certainly read.<br>Nothing comes more welcome than a<br>book to read in these days. That<br>reminds me, I dont think I ever said<br>how much I liked another book<br>William sent it me. I have forgotten, as<br>usual the name, by an old man, in the<br>manner (with differences) of Jane<br>Austen. Anyhow, I liked it, and hope it reached the public; though I suppose not.<br>I was so glad to see you again; and<br>hope the war will not make another<br>meeting too difficult.<br>Yrs sincerely	0
**54**	**1**	12/05/1940	28/03/1941	320	Dear Ben,<br>I just missed you the other day, and<br>did not know how to get hold of you.<br>Will you dine with us at 37<br>[Mecklenburgh Square] on Thursday<br>23rd? at 8. We shall be up that week,<br>and it would be very nice to see you in<br>any clothes.<br>We come up every other week,<br>normally, and any time youre in our<br>neighbourhood, please come without waiting to be asked.<br>I wonder how you like your job.<br>Yr ever	0
**55**	**1**	07/05/1935	28/03/1941	2152	Here we are in the middle of Holland.<br>So far it has been perfect blazing sun,<br>until today no accidents, except killing<br>one hen, but it was the hens fault. It is<br>extremely difficult driving however, as<br>the streets are very narrow, and there<br>are millions of cyclists like flocks of<br>swallows, and innumerable racing cars.<br>Even Cousin Thea would cycle if she<br>were a dutchwoman. We have been to<br>Amsterdam, Dordrecht, Zutphen and Haarlem. Its all next door, I mean<br>towns are only across 6 fields. The great<br>point about it is the beauty of the<br>architecture; and the awnings, which<br>are all colours, and the canals, and the<br>tulips, and flowering trees, weeping<br>their reflections into the water can<br>such a thing be said? I am so cold, and<br>my face burns like a flayed herring. I<br>can hardly write sense. We are so burnt<br>it is hardly nice to dine with an English<br>clergyman. The only Englishman we<br>have seen by the way. I have also seen<br>some of the best Rembrandts in the<br>world; and Vermeers, but you wont<br>want descriptions. But I dont see how<br>to avoid them, as we are cut off from civilisation completely. I have just got 3<br>letters, one from Ka [Arnold-Forster],<br>to ask me to visit her in a nursing<br>home, one from Violet Dickinson, to	0

**56**	**1**	08/05/1935	28/03/1941	2151	I doubt that any remarks of mine about<br>B and P have any value, as I have been<br>half asleep in the evenings and read it<br>in between great gulps of somnolence<br>So this is only an impression: that is, I<br>dont care as much for the Beecham as<br>for some of your characters, the reason<br>being, I think, that you hold your hand<br>from those incisions that cut deep,<br>because the fish is alive that youre<br>skinning. Thus I feel you skirmish<br>round and toss balls in the air, and dont settle down and pull up your<br>sleeves, but thats the inevitable<br>drawback with a living subject. And its<br>very lively and spirited, what I miss is<br>the innards. But I have only read it once,<br>and in gulps as I say. The Pharaohs<br>seemed to me to strike more boldly<br>and, directly and I enjoyed the desert<br>and the little snatches of travellers<br>figures seen against the hotel lounge<br>greatly. There you seem to swing out<br>free and come down hard. The B. as I<br>say had for me rather the air of skating<br>and flaunting where, with my passion<br>for fact, I wanted then Sir T: [Thomas<br>Beecham] unlaced his boots and went<br>to bed with her, I found the same slipperiness<br>overcame me when I wrote about<br>Sickert, But I will read again and no	0
**57**	**1**	08/05/1935	28/03/1941	2151	My dear Ka,<br>Your letter has just reached me, this<br>is the last town in Holland, and<br>tomorrow we cross into Germany and<br>drive down to Rome, so I am afraid<br>there is not much chance of seeing you.<br>We shant be back till the 1st of June. I<br>hope it does not mean that you are having<br>an operation or something: but the<br>address sounds suspicious. Let me<br>know if you are in London in June.<br>What a woman you are for these nursing homes, but I hope its not<br>much. Bruins ought to keep to the hills<br>and the rocks: not Weymouth St. W.1.<br>About Gwen [Raverat] and The<br>Land, it does not rest with us, we only<br>had the right to include it with the<br>other poems. Heinemann publishes it;<br>but the simplest thing would be for<br>Gwen to write straight to Vita,<br>Sissinghurst Castle, Kent. I rather think<br>there was an illustrated edition, but I am<br>not sure. Ia m writing in a room full of old<br>Dutch officers, listening to the loud<br>speaker, so cant spell or think. But we<br>are enjoying ourselves immensely. oh<br>what a mercy to be out of reach of London: and Holland is full of cows,<br>and canals and houses that were built<br>500 years ago and are precisely the<br>same with old ladies combing their cats	0

**58**	**1**	12/05/1935	28/03/1941	2147	Dear Stephen,<br>Your book arrived just as we were<br>making ready to start on our travels.<br>(We have been cruising about in Holland<br>and Germany and are about to cross<br>into Italy and come to rest in Rome) so<br>we didnt read it, but we will when we<br>get back. It was very nice of you to send<br>it, and especially as I cant remember<br>saying anything about Henry James.<br>There is lots to say though; and I expect<br>you have said many things Ive never thought of. He loomed up in my young<br>days almost to the obstruction of his<br>works. I am writing in a hotel lounge, half<br>asleep after 10 hours motoring. There is<br>also a great deal to say about Germany.<br>But again sleep forbids. We almost met<br>Hitler face to face.<br>I will try to remember what I thought<br>about Vienna: but do you think criticism<br>is any use? If so, why? I mean of the<br>living, by the living?<br>We shall be back in June. Leonard<br>sends his love. He drives with the<br>marmozet on his neck. And all the<br>children cried Hail! as we passed.	0
**59**	**1**	13/05/1935	28/03/1941	2146	We have just arrived, and found your<br>letter. Would you be so angelic as to<br>take 2 single rooms for us at The<br>Albergo d Inghilterra [Rome] from the<br>16th for one week. That seems best on<br>the whole, If one of the rooms could<br>have a bath, we are prepared to pay 20<br>lira a day extra If you cant get them,<br>would you get them somewhere else,<br>and leave a note at the Inghilterra,<br>saying where. We will come round to<br>you some time late in the afternoon. I will keep my news till then. In fact I am<br>so sleepy with driving over the Brenner<br>and so drunk with a bottle of wine I<br>cant write.<br>I hope you have got your passports.<br>Please make out a list of all to be done<br>and seen in a week. Its been grilling hot<br>coming across Germany, till the last<br>day, when it rained, and its raining<br>here at Verona.<br>It will be a great treat to see you, but<br>dont bother about us, as we shall have<br>plenty to see.<br>No, I cant speak any more Italian<br>but I can still read.	0

**60**	**1**	28/05/1935	28/03/1941	2131	Dear Victoria,<br>I got your letter in Pisa, (we are<br>driving home from Rome where we have<br>been staying with my sister) and hastily<br>write, in this hotel bedroom by a vile<br>light, to say we cant think why you<br>havent heard from the Hogarth Press<br>about A Room of Ones Own. Leonard<br>says they wrote long ago to Madrid. But<br>he will look into it when we go back next<br>week. I nee dont say that I shall be<br>delighted if you find you can do it. The Press will write again.<br>We have been in Holland, Germany<br>and Italy, and seen ever so many<br>different civilisations; in fact my brain is<br>so crowded I want to subside into a<br>coma, like a spinning top and cease<br>spinning. Alas, though, I have used all my<br>holiday this year, and shant get as far as<br>South America. Another time? Yes, I<br>hope so. I am still imagining vast yellow<br>butterflies and your room and the<br>flowers. And I have forgotten the address!<br>But rather than wait to get it in London<br>I will send this illegibile scrawl to the<br>English Minister in Buenos Aires and<br>trust to him to forward. And dont forget me either.	0
**61**	**1**	06/10/1935	28/03/1941	2000	Dear Lyn, We have just got back to London after rather a distracted summer. At least it ended with the Labour party at Brighton and began with Germany and Italy. Did we tell you how the marmoset saved us from Hitler? We are so glad that you are going to have a baby, and hope to see you both this autumn. Where is Comberton? I have no notion. Grantchester I know and Madingley, but thats all. Leonard is in a great rush with politics as you can imagine, and we are both at the moment regretting Monks House, which we left almost flooded, but very lovely in its ruined way. The storm turned half of every tree deep brown. Well, we shall hope for news of you, and shall turn up when we're travelling our books if we may. Now by the way, I have a niece at Newnham: a very charming niece, Ann Stephen. Leonard sends his love and congratulations.	0

**62**	**1**	08/10/1935	28/03/1941	1998		I have a thousand apologies to make, but no MS.! We had a comb out through every jungle before leaving Monks House, and found nothing. What is most likely is that I stuffed it between the leaves of a huge MS. novel I was reading and rejecting, and that it was sent back to some infuriated author who kept it in revenge. At the same time I lost a letter I had to answer, containing not mere praise but 3 pocket handkerchiefs. You must tell me faithfully what the bill for retyping is. I am getting so casual and submerged in things all these Fry papers I lose something daily. The theory is that I am so careful of the Fry letters, all my care is spent thats what I say. I heard from Vera Brittain, a heart broken letter poor woman. And I am told (not by her) that what killed poor Winifred was first an African germ, which they thought was cured; then Vera Bs father jumped into the Thames and drowned himself; Vera and W. spent several days searching for the body; found it; Vera broke down thereupon; Winifred was sent to look after the children; suddenly the germ revived; she was too exhausted to struggle, and so died; but this comes only second hand. I hope to keep freer next week than this: Athos is out; R.B. has written fairly mildly in answer to a sound drubbing by L: all the bookshops praise the set up; and first sales not bad. I mean advance sales, but we must wait. I had to send the Ladies of Ll: back to the hermaphrodite. I cant repeat my reasons on this slip; but perhaps, she will tell you. I thought it quite well done in its way.	0
**63**	**1**	25/10/1935	28/03/1941	1981		Dear Mr Ould, I am in receipt of your letter of the 25th inst. I have had no communication from Mr Ellis Roberts and I do not know therefore why he has informed you, as you say, that I have agreed to join the P.E.N. For reasons with which I need not trouble you I am unable to join the Club, and it is therefore impossible for me to sign the resolution which you have so kindly forwarded to me. I need not say how much I appreciate the kindness of the Committee in making the suggestion.	0
**64**	**1**	03/11/1935	28/03/1941	1972		Dearest Ottoline, I wish I could come, but I am afraid I cant on Tuesday. I have promised to be in to see a dull little man, and I dont suppose he will go in time. But may I come another day, evening rather, when you are alone, if you are not too busy? We are just back from Rodmell, where we had Labour Party meetings in the schoolroom not a chance of getting in of course. Give the great poet [Yeats] my humble duty, and thank you for asking me.	0
**65**	**1**	06/01/1932	28/03/1941	3369		Here are Es letters. My word are you travelling today? But this kind of remark is no pleasure in a letter, and I have written so many as its useless walking, that I cant go on even to you. I am better though, oh yes: I wrote this morning: a vast plate of saddle of mutton did the trick: I will tell you the story some time. Is it any good for your pain, plain roast meat in masses? Well Lytton is improving, and we go back [to London] on Sunday I think and I hope this blasted season of misery is more or less over.	0
**66**	**1**	13/01/1932	28/03/1941	3362		The news is very bad about Lytton tonight, though not quite hopeless. We are going to Hungerford early tomorrow, as they think it may be some help, I shall come back on Friday perhaps late tomorrow. I am much better so there is not the least risk for me. Take care of yourself.	0

**67**	**1**	14/01/1932	28/03/1941	3361	We are just back from Hamspray, so I thought you might like to hear. Lytton is better again, though they thought he was dying on Sunday. In fact the Dr. said it was hopeless, but he suddenly got better like last time [December, 1931]. He is now fearfully weak, but not actually losing strength. They have got a new specialist [Sir Arthur Hurst] who thinks the disease is running its course, but cant say how long it will be.<br>Nobody has ever seen a case like it and nothing goes as they expect. We took Pippa out and had tea with Carrington Ralph and Frances at Hamspray. They all seem worn down, but inclined to be hopeful again. Except for the general feeling that nobody knows what may happen. Lytton is conscious and determined to do all he can, and they said he liked our coming. His temp. went down to 99 this afternoon. Perhaps you have heard from James [Strachey]. We shall be here this week end.	0	
**68**	**1**	15/01/1932	28/03/1941	3360	Dearest Ottoline, Yes, I feel hopeless about ever seeing you partly I have been away and retired from life into obscurity then Lyttons illness. We were at Hungerford yesterday, and saw them all. He is desperately ill, but they think there is some hope, he recovered again, when they thought him dying on Sunday. Pippa [Strachey] is with him all the time, and says he is wonderfully<br>composed and does all he can to help. But what an awful time they are having and nobody seems to know how long the illness lasts, and what course it takes. I wish I could come next Friday, but I think we go to Rodmell, and perhaps to Hungerford again. Might I come one day the week after? I should so much like to. London is a handfull here I am interrupted and made to write nonsense by William Plomer.	0	
**69**	**1**	21/01/1932	28/03/1941	3354	Darling Pippa, I sit thinking of Lytton and Thoby and how Lytton came to me when Thoby died and I feel more than ever your sister now, darling Pippa, if you will let me. You know how we loved him.	0	
**70**	**1**	26/01/1932	28/03/1941	3349	Well, I did not write, but was grateful for your note and rest secure on your understanding my silence and all the rest. As you may suppose, I have been rather involved in the usual miseries of this sort of occasion Lord, how people suffer, and how human beings torture each other unnecessarily. But I cant go into this now: and its all over, not the tortures, no, but after this week, I suppose we shall be again as usual. I have<br>had to see a good many people, one way and another. How are you? Well, dont bother to write if you are as I am so often, in the dumps: but I should like to hear that youre not stiffening or sickening or anything horrid.	0	
**71**	**1**	02/01/1930	28/03/1941	4103	Dearest Ottoline I meant to answer your letter in London it was a very nice one; but O Lord youre mistaken if you think I enjoy what you call being surrounded by admirers. What it means is that I am pinned down in my drawing room when I want to be wandering the streets to talk to some earnest American, or summoned to the bedside of Lady Cunard where all I get out of it is the wonder of her golden silk stockings. Surely, in our time something better than this seeing people might be contrived. But you will have forgotten the sentence that roused this cry of rage in me. Here in the country one loses a little the old match box feeling the rubbed and scratched match box feeling. But I am nearly driven to set up my house in a pine wood in France. This leads, inconsistently, to saying that if you, who are so much more modest than you should be, still think it nice to see and be seen, will you come in on Wednesday night (8th I think) after dinner when you will see Goldie Dickinson, a young man called Sprott,<br>another called something else, and the semblance because I certainly shant be myself of your irascible but faithful Virginia We only skip about in old clothes as you know. However I like to see you in your splendour.	0	

**72**	**1**	01/01/1929	28/03/1941	4469	Dear Miss Tyler,<br>It is very good of you to write and tell<br>me that you like my book A room of<br>ones Own. It was the product of a<br>highly unscientific mind, and I am<br>therefore glad that you still find<br>something true in it.<br>My knowledge of Greek history is<br>small; but I suppose that writing poetry<br>was, in one island and for a certain<br>group of women, the habit at a certain period, and that Sappho was not a<br>unique writer but supported by many<br>other poetesses. That I think until the<br>late eighteenth century was never the<br>case in England. Why Sappho and the<br>others were allowed to write, I do not<br>of course presume to say. Historians<br>perhaps might help. With thanks.<br>Yours sincerely	0
**73**	**1**	03/01/1930	28/03/1941	4102	My dear Morgan,<br>Many thanks for your letter. I think<br>the only thing to do is to leave it<br>entirely to Stock [French publishers]<br>they are very critical and have turned<br>down several translators, and so far<br>seem to give Mauron the preference<br>over others. I never caught the<br>Frenchmans name, somebody in the<br>city, a friend of William Plomers. His<br>point was, having been to a lecture of<br>Maurons, that he talked bad French,<br>had a bad accent, and was not on the<br>strength of the lecture, intelligent. The<br>other frogs were some of Clives friends<br>in Paris who remarked how well Mrs<br>Dalloway was translated compared with<br>the Passage, which they professed to<br>find very bad: but who they were I dont<br>know. Raymonds [Mortimer] evidence<br>was of the same kind. Why, I wonder,<br>this hostility to Mauron in Paris? Is it<br>disinterested criticism, or is there some<br>motive behind? I shall tell Stock that I<br>have no views, and will abide by them.<br>From their letter to me, I did not realise<br>that they had already commissioned<br>Mauron.<br>Talking of Professors, do you know<br>one who would like to follow Blunden	0

**74**	**1**	12/01/1930	28/03/1941	4093	Dearest Ethel,<br>Lytton, of course, has not answered,<br>but all the same I do not think I will come:<br>the thing is I am dining out, or having<br>tea or some intolerable horror out of<br>the past here so often next week that I<br>am cross and distracted and should<br>only be a blot on your table. And I like<br>to be a radiance, as you know. So may I<br>come another time, or you come to me<br>here? How do you keep your suavity<br>and adorability, seeing all the people<br>you see I can not think.<br>My tongue dies, my heart crackles: but my love for you remains.	0
**75**	**1**	27/01/1930	28/03/1941	4078	My dear Desmond,<br>It was a great delight to read your<br>article. I never thought you would like<br>that book, and perhaps you did not: but<br>anyway you managed to write a most<br>charming article, which gave me a great<br>and unexpected pleasure. (Apart from<br>that, you must let me collect your<br>articles. This is no joke.)<br>By the way, did you refer to<br>Lawrence? The novelist marked by an<br>initial? He was not in my upper mind;<br>but no doubt was in the lower.<br>Ever so many thanks and I pray we<br>may meet when Lord Buckmaster no<br>longer asks me if I knew the late Lord<br>Tennyson.<br>Yrs affect.<br>Virginia	0
**76**	**1**	30/01/1930	28/03/1941	4075	Dear Miss Smyth,<br>If you only knew how often I have<br>wanted to write to you and only<br>did not for fear of boring you to thank<br>you for your books and articles and to<br>ask you about my great grandfather<br>Pattle who shot up out of a barrel, as<br>you say, in the Indian ocean then you<br>would not apologise. There is nothing I<br>should like better than to see you and<br>you might like me. Who knows?<br>Thursdays my husband, whom you<br>would like, is here but as you only<br>come sometimes, please say which day<br>suits you and we will keep it.<br>I am very glad you liked my little<br>book [A Room]. It was rather a wild<br>venture, but if you think there is<br>something in it, I am satisfied.<br>Yours sincerely and with as much<br>admiration as you will accept,<br>Virginia Woolf	0

**77**	**1**	31/01/1930	28/03/1941	4074	Dear William,<br>Its very good of the Oxford Society to<br>ask me but nothing will induce me to<br>open my mouth in public so long as I<br>live. I loathe lectures to hear or to give<br>and how any rational person can<br>think otherwise God knows. Can you<br>put this politely, without mitigating the<br>truth?<br>We have won our case [against the<br>Imperial Hotel Company], did we tell<br>you? they want to settle it and pay<br>expenses and screw all windows: a<br>triumph.	0
**78**	**1**	03/02/1930	28/03/1941	4071	Dearest Sibyl,<br>The wretched Wolves cant think how<br>to express their sorrow at your illness.<br>At all times unmannerly, they are at<br>their worst when they wish to show<br>their affection. Are there any books that<br>we could send? Leonard would like to<br>give you a primula, grown from seed in<br>his glasshouse. But these are nothing<br>compared with the flu, which is only to<br>be met by complete quiescence no<br>luncheon, tea or dinner. Do not allow<br>Jacques Blanche inside the house.<br>I saw Sir Arthur [Colefax] at<br>Burlington House on Tuesday, but was<br>too conscious of the distinction of the<br>lady in the chair [Edith Sitwell] to go up<br>and ask after you. Lord! there were a<br>lot of people, and many who had<br>vowed never to meet again meeting<br>there. How does one refuse the M. of<br>Londonderry? Are not I rising in the<br>scale! It is true only so far as the party to<br>which every plumbers wife in London<br>is asked, but still its rising. And I am<br>plunged into the arms of Ethel Smyth. I<br>feel them already hugging tight. It is a<br>breathless rapture. And Vanessa has a for next week. Please be careful.<br>show tomorrow; and all By is much<br>excited. And we are asked to sell this	0
**79**	**1**	04/02/1930	28/03/1941	4070	Dear Dame Ethel Smyth (I am afraid I<br>miscalled you before)<br>Alas, we are going down to the<br>country early on Friday. But if you<br>would name any day next week, I<br>would keep it free. Im sending you a<br>book of pictures by a great Aunt of<br>mine, in which I quote your opinion of<br>my great grandfather [Pattle]. But this<br>is no return whatever for the immense<br>pleasure I have had from your books (I<br>dare not say music, because though<br>willing, I am ignorant) in which my<br>husband agrees with me.	0

**80**	**1**	08/02/1930	28/03/1941	4066	Dearest Ottoline<br>(Armada is a very good name too).<br>We are down here, freezing and<br>withered in the east wind. But its very<br>beautiful too, all covered with snow,<br>pink and violet. I shall be back in a day<br>or two, but life has heaped up so many<br>muddles next week that I am rather in<br>difficulties. The week after? A Tuesday<br>or Wednesday between 5 and 6? And<br>alone or with your Lady, whom I met<br>years ago, as you like. Send a card if<br>you wish Im so unhappy about<br>Charlie. We got a desperate account as<br>we came here, only from a maid Yr VW	0
**81**	**1**	09/02/1930	28/03/1941	4065	My dear Dora,<br>I hope you will not mind my writing to<br>you it is only to gratify my own<br>feelings. I have been thinking again and<br>again at Rodmell of you and Charlie<br>and wishing I could tell you how much<br>your and his friendship has been to me.<br>It began years ago, after my brother<br>died, and all this time I have felt him<br>there, with his extraordinary goodness<br>and understanding. I do not suppose he<br>ever knew how grateful one was. And<br>yet, it is no exaggeration one will<br>need him and miss him all ones life.<br>Your letter to Leonard makes me very<br>angry with myself. How can I have<br>been such a fool as to spoil those days<br>with merciless chaff? It must have<br>been some idiotic mood probably<br>nervousness on my part. I do hope<br>you will forgive me and believe in the<br>sincerity of my affection.<br>We both send our love to you and<br>Daphne. I hope she will think of us as<br>friends (though so old) and come and see us sometimes.	0

**82**	**1**	11/02/1930	28/03/1941	4063	My dear dame Ethel Smyth.<br>If you knew how many lies I told all<br>Sunday and yesterday about not having<br>a temperature in order not to put you<br>off, and then was caught out, and then<br>said very likely you wouldnt catch it,<br>and was then forced to ring up and<br>explain, you would not accuse me of<br>telling lies anyhow to you I was<br>never so truthful in my life. It did seem<br>rather monstrous to let you come here<br>and get the influenza at your first<br>encounter: But what about Friday 4.30?<br>I expect to be all right then; or Monday<br>4.30? I will keep both till I hear.<br>And I have a request. I went to get<br>Impressions that Remain today, and<br>found only vol 2. which I have read<br>from end to end lying in front of the<br>fire with my dog. But I cant leave you<br>half in half Could you LEND me vol<br>1? It would be an angelic charity if<br>you dont hate doing up parcels. What a<br>fascinating book! How did you learn to<br>write like that?<br>Yours very sincerely<br>Virginia Woolf<br>My husband is up again but says he is stupid as an owl.	0
**83**	**1**	11/02/1930	28/03/1941	4063	Oh damn, I came back from Rodmell<br>with the flu and am in bed so I am afraid<br>I cant dine on Thursday. I suppose you<br>would not come to tea tomorrow? only<br>if I am normal though (Leonards had<br>it too). But you shant run any risk.<br>Perhaps you would ring up. It would be<br>very nice to see you.<br>Yr	0

**84**	**1**	13/02/1930	28/03/1941	4061	My dear Dame Ethel Smyth,<br>But this generosity is absolutely<br>unheard of and wrong. I said LEND<br>and instantly two volumes [of<br>Impressions that Remain] are shot at<br>my feet. Well, if you will write my<br>name in them, I can not ask more or<br>resist. Of course I read them the year<br>they came out, with rapture, and<br>bought the red Edition later, which my<br>sister Vanessa, who has only read 3<br>books in her life, stole, leaving me only<br>the second volume. I think I could<br>stand an examination in all your aunts,<br>uncles, horses and dogs. I am now rereading,<br>with the additional delight of<br>being the possessor, though I fear by<br>unfair means. It is one of my favourite<br>works, and I have even gone so far as to<br>say so in print. Yes, I think your mother<br>adorable. So was mine.<br>What a relief to my mind that you<br>are shabby! Then I need not mend the<br>hole in my solitary dress, as I had fully<br>intended. I am still in bed, and suppose should<br>have had, in honesty, to put you off<br>tomorrow, owing to a temperature; so<br>Monday will be perfect.<br>Really I cant thank you enough for the books, to which I am now going to<br>return.	0
**85**	**1**	13/02/1930	28/03/1941	4061	Look, Potto has written you this<br>cheque. Its the only possible, and most<br>painless way. Have you got it? (I mean<br>influenza). I shall be alone to tea<br>tomorrow, wh. is Friday, and, if you<br>liked to come, wd. not ask anybody.<br>Perhaps you had ring up. But for the<br>Lords sake do not bother about it. I<br>mean, I can get somebody else not as<br>nice though.<br>V.	0

**86**	**1**	14/02/1930	28/03/1941	4060	Dear Dame Ethel Smyth.<br>You will hate the sight of my<br>handwriting. Honesty, which I loathe<br>more and more, compels me to say that<br>I am still in bed with a temperature,<br>and the dr. says I am not to see anyone<br>till Im normal, and thinks there is no<br>chance of this by Monday.<br>I am so disheartened I do not know<br>what to suggest. May I let you know<br>when I am well on the chance that you<br>can come? There is nothing I should<br>like more than to see you; it is<br>infuriating to have got ill at this<br>moment. All I can do is to read you<br>and wish to goodness you had written<br>10 volumes not 2.<br>Well, I shall try for a later day next<br>week, and please do not forget your<br>promise in the meantime<br>Yrs sincerely<br>Virginia Woolf	0
**87**	**1**	16/02/1930	28/03/1941	4058	Dearest Clive,<br>What I should like would be a long<br>long letter of affection and gossip. I<br>have been in bed a week with<br>influenza, and [Dr] Elly [Rendel]<br>proposes to keep me on the sofa<br>another week. You realise therefore my<br>state of mind: and you sit naked in the<br>sun!<br>I am strongly tempted to fly to my<br>pinewood at Cassis. If so, we may meet.<br>London is too cold, too crowded, too<br>full of funerals and influenzas.<br>I am reading Byron, well, there is a<br>lot to be said about Byron. Maurois<br>does not understand him, but has the<br>merit of making me think that I do.<br>Enough though: this is only a plea for<br>a letter.<br>And hiss in Eddys ears the words<br>Virginia Isham.<br>yr	0

**88**	**1**	17/02/1930	28/03/1941	4057	My dear Dame Ethel<br>Once more.<br>I suggest this very next Thursday for<br>the following reasons.<br>The temperature is now only a small<br>one (I have often had them and they do not<br>matter).<br>I am not infectious.<br>You wont mind if I wear an old<br>dressing gown.<br>You will excuse my stupidity and put<br>it down to influenza.<br>If you dont come then, we may go<br>away, and never meet for years.<br>Also, I can telephone on Thursday<br>a.m. if anything terrible happens.<br>So I shall expect you on Thursday<br>4.30. Is this explicit?<br>Of course I want your book. I think I<br>missed that, though everything else, as I<br>believe, of yours is known to me. You<br>are, as I hope to explain a highly<br>interesting portent to us old hacks,<br>Yours sincerely<br>Virginia Woolf	0
**89**	**1**	24/02/1930	28/03/1941	4050	I liked your extravagant telegram<br>immensely and the book came and<br>your letter. Shall I get another letter?<br>Not unless I write one, perhaps. The<br>truth is that I went out, inspirited by<br>your visit, for a walk on Saturday and<br>went into the question of church<br>decoration at Hampstead garden<br>suburb, and so, not unnaturally, had to<br>retire to bed again, where I am. But this<br>time it is what they call nerve<br>exhaustion and not a temperature, if<br>that conveys anything to you.<br>It is incredible stupidity and<br>drowsiness. If I drop my book I do not<br>pick it up. This explains why, except<br>for 2 or 3 paragraphs, I have read none<br>of the Prisoner. But one of these<br>paragraphs was so interesting that I<br>thought I must have written it myself.<br>This is the highest compliment I can<br>pay any writer:<br>And that reminds me you<br>threatened to read my books. Please<br>do not. I feel without doubt and without<br>sorrow, but serenely and certainly that<br>you will not like them; and that this is not<br>one atom to your discredit or mine. It is<br>merely a matter of blue eyes or brown.<br>Let us bury my pen, and never mention	0

**90**	**1**	11/01/1929	28/03/1941	4459	I wrote in such a hurry the other night that I expect I failed to explain that Leonard and I arrive at the Friederichstrasse station on Thursday at 5.21. and shall go to the Prinz Albrecht [Hotel] if we dont see you. Could you possibly let us have a line to say what street it is on, as its not marked in the Baedeker.<br>I see that one out of every 15 people has influenza in Berlin, so I am arranging to catch it on the last day and stay on.<br>Lord! What unhappy letters you do write from Berlin!<br>V.<br>Let me know if you want anything brought.	0
**91**	**1**	14/01/1929	28/03/1941	4456	Dear Miss McAfee,<br>I am afraid that there is no chance of my being able to send you the article on Dr Burney by the tenth of February. I am only now beginning to work at it, and as I have other work to do at the same time I shall certainly not have finished it by then. Also I think it will be a good deal longer than I supposed. Would it not be as well to give up the idea, and I will find a home for it elsewhere?<br>Thank you so much for sending me the press cutting about Orlando. It was very good of you to write so kindly, and I am delighted to think that you enjoyed the book.<br>With kind regards from myself and my husband.<br>Yours very sincerely	0
**92**	**1**	27/01/1929	28/03/1941	4443	Well, here I am in bed. I had to be hauled out of my berth at Harwich a mixture of the somnifeine. Somnifène, flu, and headache apparently. Quite drugged. But I am better. Only of course the dr. makes me stay in bed and do nothing. I wish it had happened in Berlin. I wish I could see you. Do write.<br>I am much better today. Berlin was quite worth it anyhow.<br>Love,<br>Virginia<br>The doctor just been says its the flue and I shall be able to get up on Tuesday.<br>VW	0

**93**	**1**	28/01/1929	28/03/1941	4442	How nice to get your letter this morning a great treat. I am afraid I wrote you rather a dazed one yesterday. That blessed sea sick drug of Nessa somehow went wrong and I had to be hauled along like a sack, but thats all right now, and so thats the flue, and I have only got the usual headache which is better today.<br>I daresay I shall get up tomorrow.<br>I am being rather strictly looked after though by Leonard and Ellie Rendel (the dr.) and so can only write these scraps. I keep thinking of you and long oh Lord how I long, that you would open the door and come in. I have seen no one, so you need have no fears. I do nothing but sleep. Pinker [spaniel] lies on the chair by the fire. Leonard comes in with a proof or the paper, Nelly brings me lemonade. I read the Times and drop it. I see you with extreme distinction. Well anyhow it was worth the week with you. I think of the tower and the lights and the waves and the shell room at Sans Souci and you, and you. Next week is Feb. 1st. so there is really not long to wait. But Lord! what a horror Berlin and diplomacy are! I'd no idea till I had seen it. And I shiver at	0
**94**	**1**	29/01/1929	28/03/1941	4441	Dearest Creature,<br>Here is another selfish invalids bulletin, but I like to write to you, and you will not mind it all being about myself.<br>I am really better today, only still kept in bed. It is merely the usual headache which is now making me rather achy and shivery but passing off. And the dr has just been and says I shall get on the sofa tomorrow and may see someone on Thursday. Who would you like to see she says, and I think at once of, well, it aint Mary [Hutchinson] or Christa: though I daresay to tease you I shall have Mary, and as I shall be in my nightgown. But no. I am very faithful. Its odd how I want you when I am ill. I think everything would be warm and happy if Vita came in. And Vitas having tea with the American Ambassadors wife, I suppose: very smart and haughty. How you frightened me that night on the pavement! Potto still talks of it.<br>Its so odd to have gone straight from all that movement, and big houses, and street signs and wine and Vita to lying alone in bed up here without any interest. I have looked out of the window	0

**95**	**1**	30/01/1929	28/03/1941	4440	Another 5 minutes conversation, all about myself as usual. Do you mind? Are you bored? You're the only person I write to.<br>I am down on the sofa in the drawing room this evening. The Knole sofa, very comfortable. I cant be bothered to find a pen so you must put up with pencil. These headaches are very odd. This time last week we were at Sans Souci, now I cant imagine walking across the Square. What do you think happens? Leonard and the dr. says its my rackety life in Berlin. But why this change in 10 seconds? I am really better, only rather cross that it takes so long. And that I cant see you. If you were to sit by me now I should be so happy. Brilliant ideas come into my head, scenes, solutions, but are extinguished. How does one write? I read half a page about Austin Dobson and then drowse for an hour. Leonard brings in huge beef steaks. I say I am afraid I shall be very strictly kept under for a time. No parties, no romances. But that suits you very well, you wretch. You want Potto and Virginia kept in their kennel, write dearest please anything that comes into your	0
**96**	**1**	01/02/1929	28/03/1941	4438	It was very refreshing to get your letter this morning, among such dreary ones. I have read it several times. Of course we would go to Long Barn like a shot you know. The dr. says though that I must be in bed another week anyhow and then be very quiet, oh this being very quiet! So that I do not suppose we could get away till the middle of Feb, and then you would soon be back. But if London becomes intolerable I shall take you at your word. At the moment I am so lethargic I dont think of anything but walking down to the sofa or up to bed. They give me stiff doses of bromide. Its rather nice. Ones head feels grown to the pillow. One floats like a log. I wake to write to you. The sight of you on [the] street at Berlin is my last record of humanity. There were two Germans in the carriage fat, greasy, the woman with broken nails. The man peeled an orange for her. She squeezed his hand. It was repulsive. Perhaps in time one might come to think this complete unadornment beautiful, morally, after many many years. You deserve Rapallo and I envy you. Heat sun anyhow clear air. Its lead and yellow here day after day. Oh	0

**97**	**1**	03/02/1929	28/03/1941	4436	My dear Julian,<br>Many happy returns! To think that<br>you are 21! And I carried you round<br>the room in my arms once.<br>We send you a small cheque to buy a<br>book or a bird or a chocolate cake,<br>whatever you like, with our blessing.<br>The trapesing in Berlin was terrific.<br>Nessa poisoned me with a seasickdraught.<br>I sank into coma and am still<br>in bed.<br>Are you writing poems? I should like<br>to see them some day.<br>Write me a long long letter.<br>Yr loving Virginia	0
**98**	**1**	07/02/1929	28/03/1941	4432	Dearest<br>I have been enjoying your letter<br>extremely. This is only to say that<br>there is no reason whatever to think it<br>was your drug that did me harm. Elly<br>says I may have had slight flue, but<br>anyhow all the blame is put on Berlin<br>and this would have happened anyhow,<br>though possibly not in such a sudden<br>way.<br>It was very odd, waking at Harwich<br>in a state of apparent intoxication. Also,<br>I took less than the proper dose, and it<br>was from the same bottle you had<br>taken.<br>I am much better and am spending<br>the day on the sofa today, and not in<br>bed. I intend to begin work on<br>Monday.<br>Elly is rather severe, and Ive only<br>seen Helen, who is thought less exciting<br>than Mary.<br>The only gossip therefore is rather<br>remote: I daresay you have heard that<br>Angus has not got the Nat. Gall. It has<br>gone to an unknown man. Miss Ritchie<br>has been to ask Leonard to help. Angus she<br>says, is now sunk into complete apathy,<br>wont try for any job, and says he means to write plays with her, six words omitted.	0

**99**	**1**	10/02/1929	28/03/1941	4429	Dear Dorothy,<br>I was very pleased to get your letter,<br>it was charming of you to write. I have<br>been in bed for over a fortnight with<br>some sort of flu that has a disastrous<br>effect upon the nervous system. Elly has<br>been giving me stiff tumblers of<br>bromide. And your letter gleamed<br>through my drowsiness like the fin of a<br>silver fish. In fact I pulled it out and<br>read it several times. But I wish I could<br>have got more into my sketch of your<br>mother. When I came to write (and it<br>had to be scribbled in a great rush) I<br>found the image of her somehow so<br>important, so predominating in my<br>mind, I was surprised, considering<br>how little I had seen of her.<br>Yes, you are an arch-flatterer. Its<br>done with an air of intense conviction<br>on top of immense erudition and<br>insight which is irresistible. I do hope<br>our annual teas arent doomed: you<br>must come to London, if for them only.<br>I would throw in 2 ices and a cocktail.<br>Tell Janie I am so glad she has begun to<br>take liberties with me and the Sphinx<br>[Leonard?]. High time she did. And<br>excuse this handwriting. I am up, but<br>write lying down.	0
**100**	**1**	04/03/1929	28/03/1941	4407	Dear Mr Hayward,<br>It was very good of you to write to<br>me about my article. I never know<br>whether these things are supposed to be<br>secret or not. Anyhow, as you have<br>guessed I am delighted to claim your<br>praise, which is very welcome. I daresay<br>one could have found out more about<br>Miss Jewsbury; I had only one volume<br>of her letters to go upon. I could not<br>read more than one of the novels, and I<br>expect that some old gentleman who<br>has read all mid-Victorian memoirs will<br>blast my theories completely. Her<br>relation with Mrs Carlyle was<br>interesting, and I had to be discreet.<br>Yours sincerely,<br>Virginia Woolf	0
