# Supplementary material for: Potential use of text classification tools as signatures of suicidal behavior: A proof-of-concept study using Virginia Woolf’s personal writings
Source: PLoS One. 2018 Oct 24;13(10):e0204820. doi: 10.1371/journal.pone.0204820 (PMC6200194; doi:10.1371/journal.pone.0204820)
Supplement: S1 Table — (PDF) [file pone.0204820.s011.pdf]

S1 Table. Words written in the last 60 days before Virginia Woolf's suicide.

Words	Frequency
the	299
and	176
you	169
but	73
this	58
she	43
all	36
what	35
one	32
will	32
now	29
cant	27
then	26
its	24
like	24
think	22
can	21
write	21
dear	20
time	20
dont	19
say	19
see	19
come	17
leonard	17
must	17
read	17
shall	17
just	15
book	14
last	14
letters	13
two	13
day	12
much	12
suppose	12
came	11
old	11
back	10
get	10
london	10

yes	10
letter	9
said	9
another	8
life	8
vita	8
find	7
miss	7
never	7
nothing	7
story	7
great	6
little	6
house	5
war	5
blue	4
books	4
love	4
