# Supplementary material for: Potential use of text classification tools as signatures of suicidal behavior: A proof-of-concept study using Virginia Woolf’s personal writings
Source: PLoS One. 2018 Oct 24;13(10):e0204820. doi: 10.1371/journal.pone.0204820 (PMC6200194; doi:10.1371/journal.pone.0204820)
Supplement: S2 Table — (PDF) [file pone.0204820.s012.pdf]

S2 Table. Different words written in the last 60 days before Virginia Woolf's suicide compared to outside of 60 days prior to Virginia Woolf's suicide.

Words	Frequency of word written in the last 60 days before Virginia Woolf's suicide	Frequency of word written outside of the 60 days prior Virginia Woolf's suicide
you	169	0
this	58	0
she	43	0
all	36	0
then	26	0
two	13	0
suppose	12	0
came	11	0
yes	10	0
said	9	0
vita	8	0
miss	7	0
story	7	0
house	5	0
war	5	0
blue	4	0
books	4	0
ask	0	14
bed	0	17
better	0	13
bed	0	17
good	0	17
got	0	17
hope	0	20
how	0	56
know	0	18
long	0	20
many	0	24
may	0	15
nice	0	14
rather	0	30
room	0	13
says	0	17
seen	0	12
tell	0	17
though	0	13

tomorrow	0	6
virginia	0	20
way	0	6
week	0	12
well	0	22
