# Supplementary material for: Potential use of text classification tools as signatures of suicidal behavior: A proof-of-concept study using Virginia Woolf’s personal writings
Source: PLoS One. 2018 Oct 24;13(10):e0204820. doi: 10.1371/journal.pone.0204820 (PMC6200194; doi:10.1371/journal.pone.0204820)
Supplement: S3 Table — (PDF) [file pone.0204820.s013.pdf]

S3 Table. Words written outside of 60 days prior to Virginia Woolf's suicide.

Words	Frequency
the	550
and	387
but	143
will	65
like	60
one	59
how	56
think	54
its	50
can	42
see	42
what	42
write	41
shall	39
come	33
dear	33
book	31
cant	31
now	31
rather	30
get	29
dont	27
great	27
must	27
leonard	26
say	26
many	24
old	24
read	24
time	24
letter	23
day	22
much	22
well	22
never	21
hope	20
long	20
virginia	20
last	19
know	18
london	18

back	17
bed	17
good	17
got	17
says	17
tell	17
another	15
may	15
ask	14
nice	14
better	13
nothing	13
room	13
though	13
love	12
seen	12
week	12
tomorrow	6
way	6
