# Supplementary material for: Potential use of text classification tools as signatures of suicidal behavior: A proof-of-concept study using Virginia Woolf’s personal writings
Source: PLoS One. 2018 Oct 24;13(10):e0204820. doi: 10.1371/journal.pone.0204820 (PMC6200194; doi:10.1371/journal.pone.0204820)
Supplement: S4 Table — (PDF) [file pone.0204820.s014.pdf]

S4 Table. Common words written in the last 60 days before Virginia Woolf's suicide.

Words	Frequency of word written in the last 60 days before Virginia Woolf's suicide	Frequency of word written outside of the 60 days prior Virginia Woolf's suicide
the	299	550
and	176	387
but	73	143
one	32	59
will	32	65
now	29	31
cant	27	31
its	24	50
like	24	60
think	22	54
can	21	42
day	21	22
write	21	41
dear	20	33
time	20	24
dont	19	27
say	19	26
see	19	42
come	17	33
leonard	17	26
must	17	27
read	17	24
shall	17	39
book	14	31
last	14	19
much	12	22
old	11	24
back	10	17
get	10	29
london	10	18
letter	9	23
another	8	15
never	7	21
nothing	7	13
great	6	27
love	4	12
